# IDD16 negatively regulates stomatal initiation via trans‐repression of *SPCH* in *Arabidopsis*

**DOI:** 10.1111/pbi.13070

**Published:** 2019-03-27

**Authors:** Shi‐Lian Qi, Qing‐Fang Lin, Xuan‐Jun Feng, Hui‐Ling Han, Jie Liu, Liu Zhang, Shuang Wu, Jie Le, Eduardo Blumwald, Xue‐Jun Hua

**Affiliations:** ^1^ Key Laboratory of Plant Resources and Beijing Botanical Garden Institute of Botany Chinese Academy of Sciences Beijing China; ^2^ College of Life Sciences Zhejiang Sci‐Tech University Hangzhou Zhejiang China; ^3^ College of Horticulture Fujian Agriculture and Forestry University Fuzhou Fujian China; ^4^ College of Crop Science Fujian Agriculture and Forestry University Fuzhou Fujian China; ^5^ Key Laboratory of Plant Molecular Physiology CAS Center for Excellence in Molecular Plant Sciences Institute of Botany Chinese Academy of Sciences Beijing China; ^6^ College of Life Sciences Fujian Agriculture and Forestry University Fuzhou Fujian China; ^7^ University of Chinese Academy of Sciences Beijing China; ^8^ Department of Plant Sciences University of California Davis CA USA

**Keywords:** IDD16, SPCH, stomatal development, water use efficiency, drought tolerance, *Arabidopsis thaliana*

## Abstract

In *Arabidopsis*, the initiation and proliferation of stomatal lineage cells is controlled by SPEECHLESS (SPCH). Phosphorylation of SPCH at the post‐translational level has been reported to regulate stomatal development. Here we report that *IDD16* acts as a negative regulator for stomatal initiation by directly regulating *SPCH* transcription. In *Arabidopsis*, *IDD16* overexpression decreased abaxial stomatal density in a dose‐dependent manner. Time course analysis revealed that the initiation of stomatal precursor cells in the *IDD16*‐OE plants was severely inhibited. Consistent with these findings, the transcription of *SPCH* was greatly repressed in the *IDD16*‐OE plants. In contrast, *IDD16‐RNAi* transgenic line resulted in enhanced stomatal density, suggesting that *IDD16* is an intrinsic regulator of stomatal development. ChIP analysis indicated that *IDD16* could directly bind to the *SPCH* promoter. Furthermore, *Arabidopsis* plants overexpressing *IDD16* exhibited significantly increased drought tolerance and higher integrated water use efficiency (WUE) due to reduction in leaf transpiration. Collectively, our results established that IDD16 negatively regulates stomatal initiation via trans‐repression of *SPCH*, and thus provide a practical tool for increasing plant WUE through the manipulation of *IDD16* expression.

## Introduction

Drought is a detrimental environmental factor that greatly affects plant growth and limits agricultural productivity worldwide. Plants can withstand water‐deficit stress via reducing transpirational water loss by altering the stomatal aperture in response to water stress (Chaerle *et al*., 2005; Chaves *et al*., 2003; Kim *et al*., 2010; Nilson and Assmann, 2007). Alternatively, altering stomatal density in response to water‐deficit stress can provide an efficient strategy to avoid tissue dehydration and improve water stress tolerance (Franks *et al*., 2015; Meng and Yao, 2015; Wang *et al*., 2016; Yoo *et al*., 2010; Yu *et al*., 2008). In *Arabidopsis thaliana*, stomata are produced through a number of successive cell state transitions and divisions. First, a subset of protodermal cells becomes meristemoid mother cells (MMC) through a mechanism that is not yet well‐understood (Pillitteri and Dong, 2013). Next, the MMC undergoes an asymmetric entry division to produce a small triangular meristemoid and a larger sister cell named a stomatal lineage ground cell (SLGC). All meristemoids have stem cell‐like character and can usually undergo 1–3 rounds of asymmetric divisions which regenerate the meristemoids and increase the total number of SLGCs. Eventually, meristemoids lose their capacity of asymmetric division and differentiate into guard mother cells (GMCs; MacAlister *et al*., 2007; Pillitteri *et al*., 2007). Then a GMC divides once symmetrically to yield two guard cells (GCs) of a stoma (Ohashi‐Ito and Bergmann, 2006).

Three basic helix‐loop‐helix (bHLH) transcription factors SPEECHLESS (SPCH), MUTE and FAMA are key switches for the successive stages in the stomatal development (Lau and Bergmann, 2012; Pillitteri and Torii, 2012). SPCH is essential for MMC formation, stomatal entry divisions, and maintenance of meristemoid identity. *spch* mutants fail to initiate stomatal lineages and produce an epidermis composed solely of pavement cells (MacAlister *et al*., 2007). MUTE is required to terminate asymmetric division and promote the transition of meristemoids to GMCs. In the *mute* mutant, meristemoids undergo excessive asymmetric divisions and create a rosette pattern in the epidermis (Pillitteri *et al*., 2007). FAMA promotes the final transition from GMC to GC. Loss of FAMA function leads to excessive GMC symmetric divisions and lack of GC differentiation, resulting in the production of long parallel rows of cells (Ohashi‐Ito and Bergmann, 2006). Two additional bHLH proteins, SCRM (ICE1) and SCRM2, act redundantly to coordinate the activities of SPCH, MUTE and FAMA through heterodimerization (Kanaoka *et al*., 2008).

As a master regulator in stomatal development, *SPCH* controls the initiation and proliferation of cells in stomatal lineage. Therefore, the expression of *SPCH* is under tight and dynamic control, which is crucial for defining stomatal pattern and density. SPCH has been shown to be regulated through phosphorylation by MITOGEN ACTIVATED PROTEIN KINASE (MAPKs), GLYCOGEN SYNTHASE 3 KINASE (GSK3) and CYCLIN DEPENDENT KINASE (CDK) families at the post‐translational level (Gudesblat *et al*., 2012; Lampard *et al*., 2009; Yang *et al*., 2014). Phosphorylation by MAPKs or GSK3 targets SPCH for degradation, leading to decreased entry divisions and ultimately a lower stomatal density (Lampard *et al*., 2009; Gudesblat *et al*., 2012). MAPKs, GSKs and CDKs upstream of SPCH are broadly expressed in the leaf, so additional signals are required in specific cells to produce the proper pattern of SPCH activity (Simmons and Bergmann, 2016). Although the phosphorylation of SPCH has been well characterized, the identity(ies) of the regulator(s) controlling *SPCH‐*mediated stomatal initiation at the transcriptional level remains elusive. Thus far, there are only a few transcription factors that have been reported to bind directly to the *SPCH* promoter region and possibly regulate its transcription. The *Arabidopsis* retinoblastoma homolog RBR1 (Retinoblastoma‐related protein 1) could interact *in vivo* with the *SPCH* promoter in a ChIP assay (Weimer *et al*., 2012). Also, it appears that SPCH could bind to its own promoter and likely form a positive feedback for its own expression (Lau *et al*., 2014). The LLM‐domain B‐GATA transcription factor GNL1 (GNC‐Like/cytokinin responsive GATA factor 1) interacted *in vivo* with the *SPCH* promoter and may be involved in red light‐induced *SPCH* expression and stomatal development in hypocotyls (Klermund *et al*., 2016).

Here, we characterized the function of a C2H2 zinc finger transcription factor of the INDETERMINATE DOMAIN (IDD) family, encoded by *AT1G25250*, which has previously reported to be involved in organ morphogenesis and gravitropic responses according to the regulation of spatial auxin accumulation, and annotated as *Arabidopsis* IDD16 (Colasanti *et al*., 2006; Cui *et al*., 2013). We showed that the *Arabidopsis* C2H2 transcription factor IDD16 acts as a negative regulator of stomatal initiation by directly regulating *SPCH* transcription. *Arabidopsis* plants overexpressing *IDD16* displayed enhanced water‐deficit stress tolerance and WUE due to reduced stomatal density caused by *SPCH* repression.

## Results

### Stomatal initiation was inhibited on leaf abaxial epidermis of *IDD16*‐OE plants

**Figure 1:**
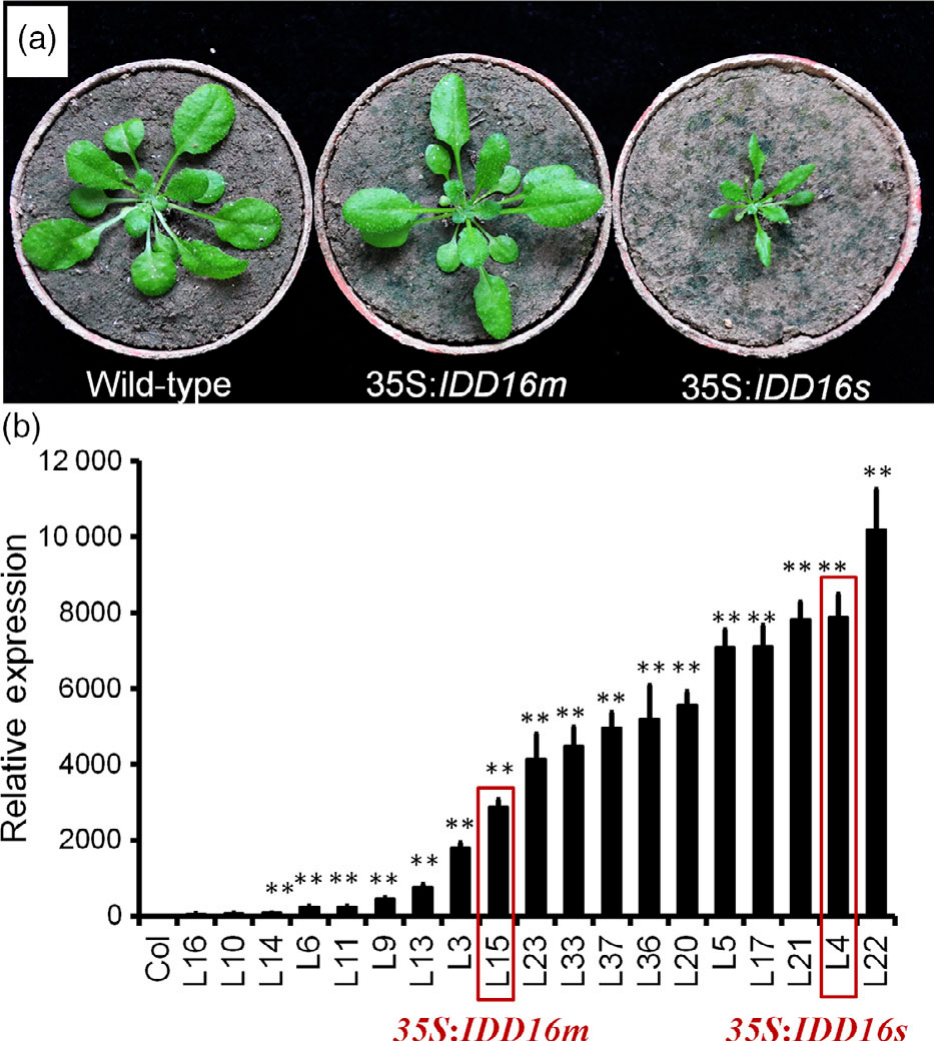
Growth phenotype of wild‐type, 35S: *IDD16m* and 35S: *IDD16s* plants. (a) Morphological phenotype of wild‐type (Col‐0), 35S: *IDD16m* (mild) and 35S: *IDD16s* (severe), which were grown in soil for 3 weeks, and representative individuals were photographed. (b) The expression level of *IDD16* in transgenic lines. qRT‐PCR was performed on total RNA from 4‐DAG wild‐type and *IDD16*‐OE plants. Values are means ± SE from three biological replicates. Asterisks indicate statistical significance based on Student's *t* test; ** *P* < 0.01.

To assess the role(s) of IDD16, we generated transgenic *Arabidopsis* plants overexpressing *IDD16* cDNA under the control of the constitutive cauliflower mosaic virus (CaMV) 35S promoter. A number of transgenic lines was obtained displaying two types of phenotypes; some lines were short in height and possessed curly leaves and some lines were relatively normal in height and displayed slightly curly leaves (Figure 1a). A similar phenotypical effect (curly leaves, short plant size) was described in transgenic plants overexpressing *IDD16* (Cui *et al*., 2013). The severity of the abnormal phenotype of the *IDD16* plants was positively correlated with *IDD16* expression levels (Figure 1b). Two representative lines, *35S:IDD16s* (severe, for highly expressing transgenic lines) and *35S:IDD16m* (moderate, for moderately expressing transgenic lines) were chosen for the rest of the experiments shown in this study.

**Figure 2:**
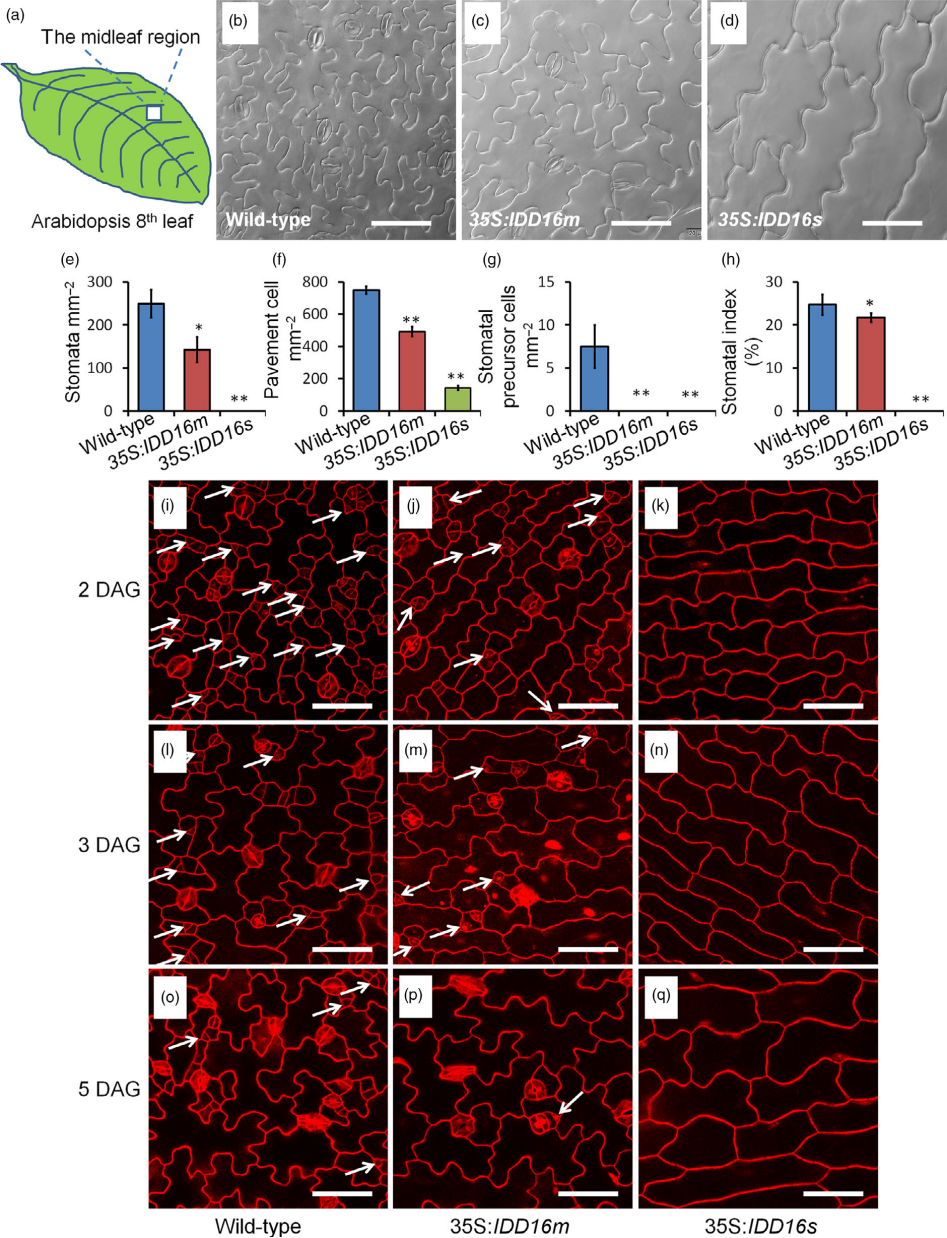
*IDD16*‐OE plants have lower stomatal densities. (a) The midleaf region of the eighth rosette leaf was used for analysing the stomatal phenotype. (b–d) Images of abaxial epidermal layers of the eighth leaf from 5‐week‐old wild‐type (Col‐0) and *IDD16*‐OE plants. (e–h) Stomata density, pavement cell density, the number of stomatal precursor cells, and stomatal index were analysed in the eighth leaf abaxial epidermal layers from wild‐type and *IDD16*‐OE plants. Three areas in the midleaf region were imaged per leaf. Data are the mean of six individual plants (mean ± SE,* n* = 6). Asterisks indicate statistical significance based on Student's *t* test. ** *P* < 0.01; * *P* < 0.05. (i–q) Confocal images of the cotyledon epidermis of wild‐type, 35S: *IDD16 m* and 35S: *IDD16s* seedlings between 2 and 5 DAG. Cell outlines were visualized by propidium iodide (red) staining and the white arrows indicate the precursor cells. Scale bars: 50 μm.

We examined the stomatal density in the midleaf region of the eighth rosette leaf from both 35S:*IDD16s* and 35S:*IDD16m* transgenic lines (Figure 2a‐d). On the abaxial side of fully expanded leaves of 35S:*IDD16m*, the stomatal density was reduced to 57% of that of the wild‐type plants (Figure 2e). More dramatically, no abaxial stomata could be seen in the 35S:*IDD16s* leaves (Figure 2d). As a result, the stomatal indices (number of stomata per total number of epidermal cells) in both lines were also decreased, albeit to different extents (Figure 2h), suggesting altered stomatal development in *IDD16*‐OE plants. We also noticed that the pavement cells were larger in the leaves of *IDD16*‐OE plants (Figure 2b‐d), which resulted in a lower pavement cell density (Figure 2f). Precursor cells could be found in leaves of the wild‐type plants but not in leaves of 35S:*IDD16m* and 35S:*IDD16s* plants (Figure 2g), suggesting that stomatal initiation was inhibited in *IDD16*‐OE plants (Bergmann and Sack, 2007; Casson and Hetherington, 2010). To confirm this assumption, the cotyledons of the seedlings were examined over a time course from 2 to 5 days after germination (DAG; Figure 2i‐q). At 2 DAG, the amount of meristemoid cells (white arrows) in the abaxial epidermis was significantly reduced in 35S:*IDD16m* (Figure 2j), compared to that in the wild‐type (Figure 2i). As shown in Figure 2k, no asymmetric entry division could be observed in 35S:*IDD16s*, suggesting that *IDD16* inhibited stomatal initiation, probably by affecting asymmetric entry divisions, reminiscent of the *spch* mutant phenotype, showing the inhibition of stomatal initiation (MacAlister *et al*., 2007; Pillitteri *et al*., 2007). Similar phenotype was observed in the cotyledons of 3‐DAG and 5‐DAG seedlings (Figure 2l‐q). In addition, the abaxial stomatal density in *IDD16*‐OE plants was negatively correlated with the level of *IDD16* transcripts, confirming that *IDD16*‐overexpression affected the stomata density in a dose‐dependent manner (Figure S1A and B).

### Effect of *IDD16* overexpression on stomatal development in the leaf adaxial epidermal layers

**Figure 3:**
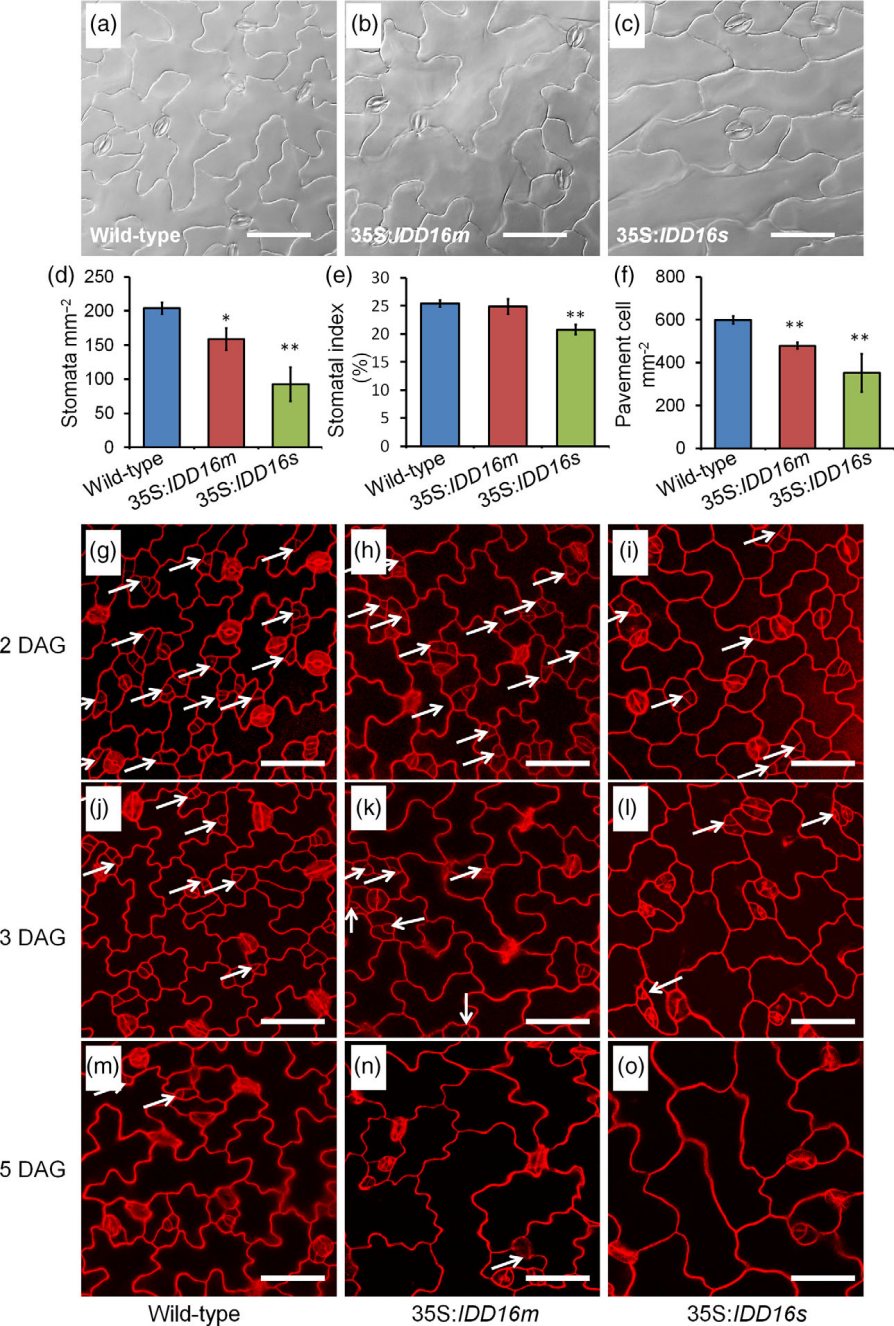
Effect of *IDD16* overexpression on stomatal development in the leaf adaxial epidermis. (a–c) Images of leaf adaxial epidermal layers of the eighth leaf from 5‐week‐old wild‐type (Col‐0) and *IDD16*‐OE plants. (d–f) Stomatal density, stomatal index and pavement cell density were analysed in the leaf adaxial epidermis of the eighth leaf from wild‐type and *IDD16*‐OE plants. Three areas in the midleaf region were imaged per leaf. Data are the mean of six individual plants (mean ± SE,* n* = 6). Asterisks indicate statistical significance based on Student's *t* test. ** *P* < 0.01; * *P* < 0.05. (g–o) Confocal images of the cotyledon adaxial epidermis of wild‐type (Col‐0), 35S: *IDD16m* and 35S: *IDD16s* seedlings between 2, 3, 5 DAG. Cell outlines were visualized by PI staining and the white arrows indicate the precursor cells. Scale bars: 50 μm.

Given the crucial roles of stomata in plant growth and development, particularly for gas exchange, the lack of stomata in the abaxial epidermis of 35S:*IDD16s* was intriguing. This prompted us to assess the stomatal development on the adaxial epidermis of the eighth rosette leaves in 35S:*IDD16s* (Figure 3a‐c). In contrast to what was observed on the leaf abaxial side, stomata could be observed on adaxial epidermis of mature leaves of 35S:*IDD16s*, although with a decreased stomatal density (45% of that of the wild‐type, Figure 3d). Similarly, the stomatal density on the adaxial epidermis of 35S:*IDD16m* leaves was reduced by 22% of that in the wild‐type leaves (Figure 3d) and less affected than the stomatal density on the abaxial epidermis (43% reduction, Figure 2e). Both the stomatal indices and the pavement cell densities on the adaxial epidermis were less decreased than the abaxial epidermis (Figure 3e and f). We examined the early stomatal development in adaxial epidermis of cotyledons (Figure 3g‐o). A considerable amount of stomata precursor cells was found in 35S:*IDD16s* (Figure 3i and l), suggesting that the asymmetric entry divisions were not completely inhibited. So, IDD16 might exert differential effects on stomata initiation in each side of the leaves.

### *IDD16* negatively modulated *SPCH* expression at transcriptional level

**Figure 4:**
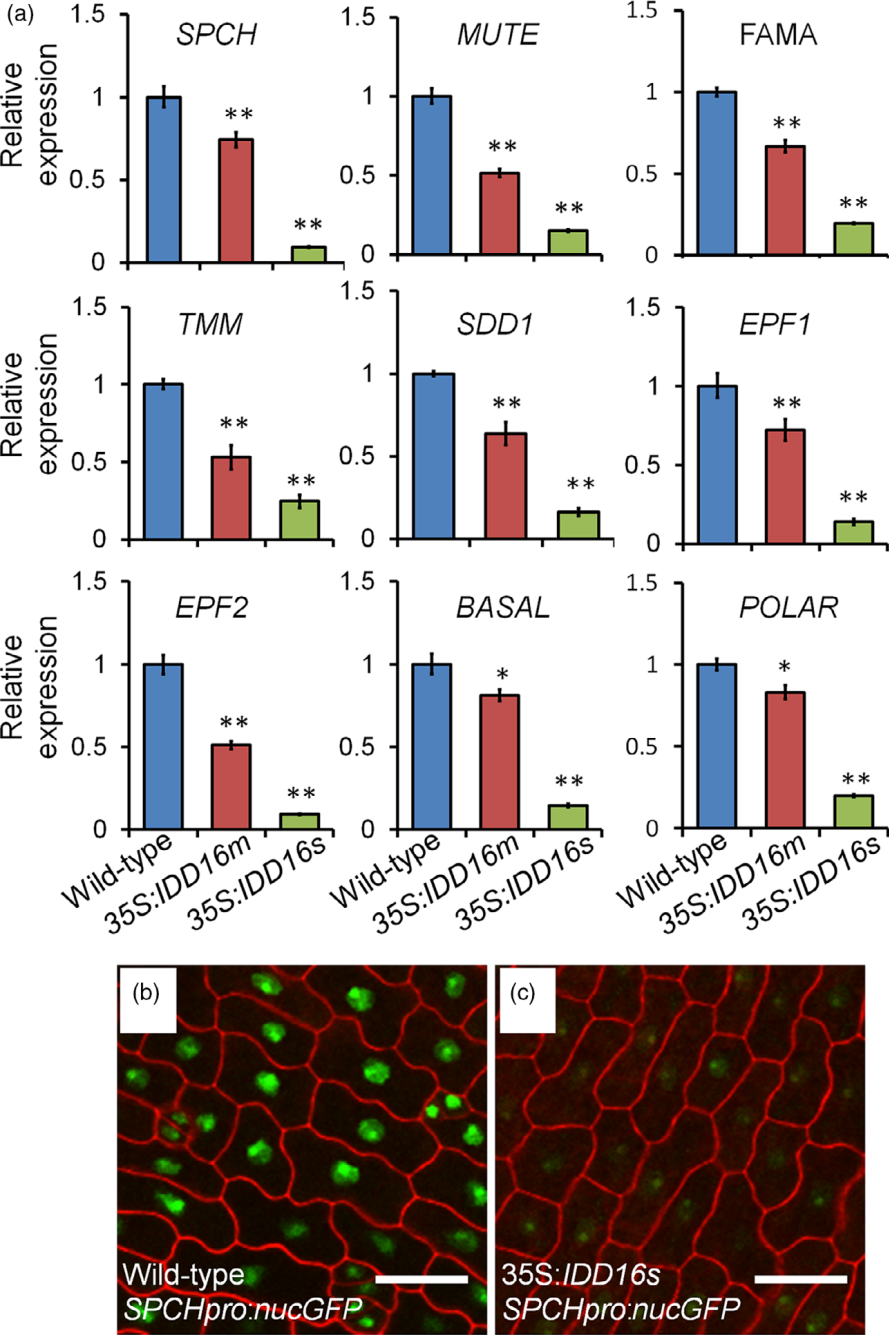
IDD16 inhibits stomatal initiation by negatively regulating *SPCH* expression. (a) Analysis of the expression of genes related to stomatal development. qRT‐PCR was performed on total RNA from 4‐DAG wild‐type and *IDD16*‐OE plants. The expression level in wild‐type was normalized as 1. Values are means ± SE from three biological replicates. Asterisks indicate statistical significance based on Student's *t* test; ** *P* < 0.01; * *P* < 0.05. (b) and (c) Confocal images of *SPCHpro:nucGFP* abaxial cotyledons in wild‐type (b) and *35S:IDD16s* (c) background, respectively. Epidermal cell periphery is highlighted by propidium iodide (red) staining. Scale bars: 25 μm.

To shed light on the molecular mechanism(s) underlying the defect(s) in stomata development of the *IDD16*‐OE plants, we assessed the expression of *SPCH* and other 20 genes associated with stomatal development in wild‐type and *IDD16‐*OE transgenic plants (Figures 4a and S2). The transcript levels of nine key regulator genes were significantly decreased in 35S:*IDD16s* and 35S*:IDD16m* plants as compared to the wild‐type (Figure 4a). Among them, *SPCH* directing the first asymmetric division that establishes the stomatal cell lineage. *spch* mutants failed to initiate stomatal lineages and all epidermal cells became pavement cells (MacAlister *et al*., 2007; Pillitteri *et al*., 2007), similar to the phenotype that we observed in abaxial leaf epidermis of 35S:*IDD16s* (Figure 2d). To discern whether the reduced *SPCH* expression was due to reduced promoter activity, we utilized *SPCHpro:nucGFP* to visualize the fluorescence produced due to the *SPCH* promoter activity in the abaxial epidermal cells of 1‐DAG cotyledons. As shown in Figure 4c, although under 35S:*IDD16s* background, the nuclear signal of *SPCHpro:nucGFP* was detected basically in every cell, the signal intensity was dramatically decreased as compared to that observed under the wild‐type background (Figure 4b), indicating that the reduced *SPCH* expression in 35S:*IDD16s* was due to a reduced activity of the *SPCH* promoter.

With regards to the other eight down‐regulated genes (Figure 4a), they are all specifically expressed in precursor cells or newly formed stomata (Pillitteri and Dong, 2013), thus upon the repression of *SPCH* transcription, stomatal initiation would be inhibited and the number of precursor cells would be fewer. For example, in contrast to the wild‐type background, *FAMA:GFP* signal in the 35S:*IDD16s* background could still be observed on the adaxial epidermis within GMC and newly formed stomata with similarly intensity to that of the wild‐type (Figure S3), even though *FAMA:GFP* expression could not be detected in the abaxial epidermis, since there were no stomata. These results indicated that the reduced *FAMA* transcript levels were due primary to a lesser amount of cells expressing *FAMA*, rather than the inhibition of *FAMA* transcription activity.

### *IDD16* is a negative regulator of stomatal initiation

**Figure 5:**
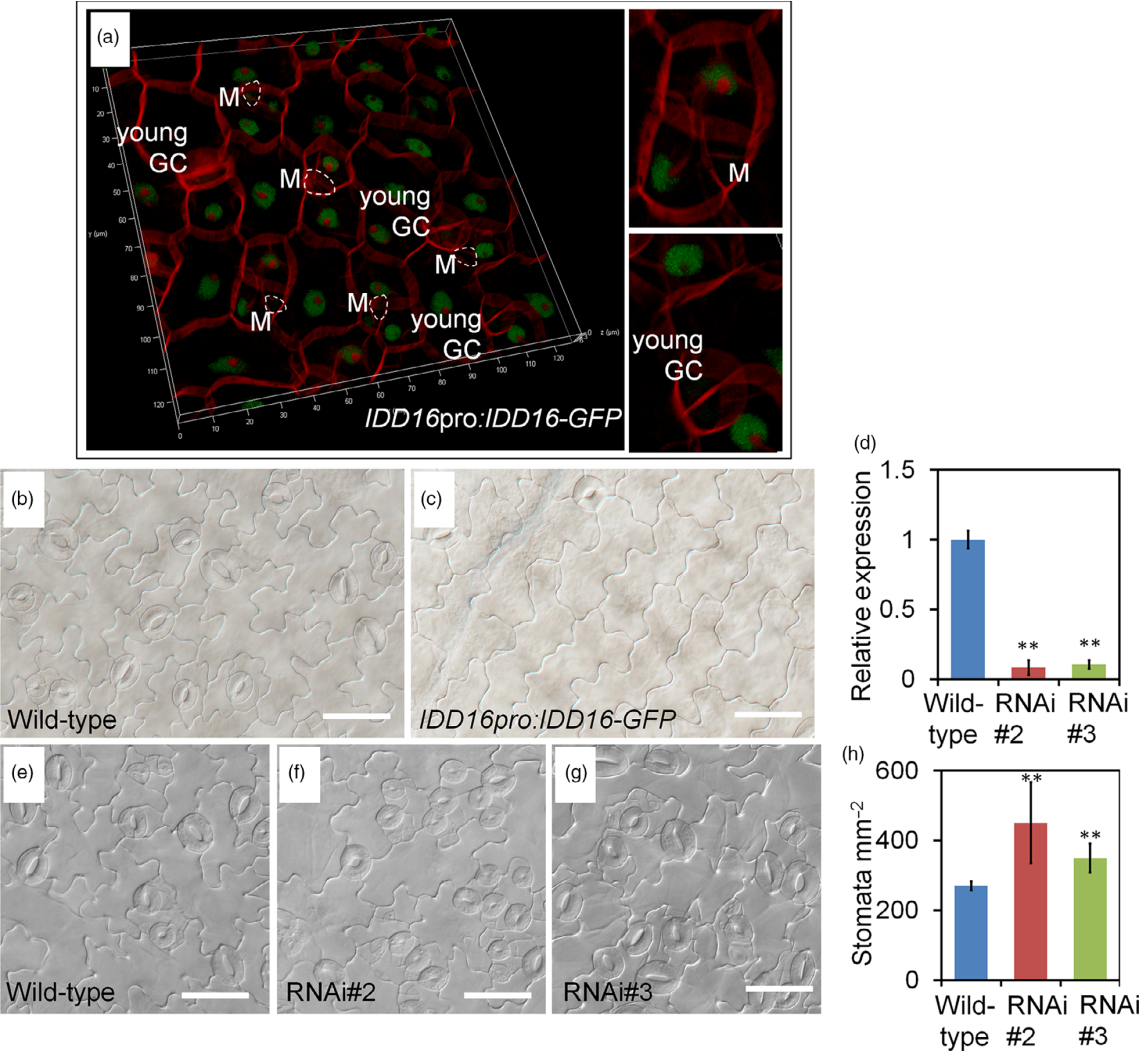
IDD16 negatively regulates stomatal development. (a) Confocal microscopy image of *IDD16pro:IDD16*‐*GFP* showing nuclear localization of IDD16 in adaxial epidermis of cotyledons in 1‐DAG seedlings. Epidermal cell periphery is visualized by propidium iodide (red) staining. White circles indicate meristemoids (Ms). (b and c) Compared to wild‐type (b), *IDD16pro:IDD16*‐*GFP* transgenic line (c) showed reduced stomatal density in abaxial epidermis of the cotyledons from 8‐DAG seedlings. Scale bars: 50 μm. (d) The expression levels of *IDD16* in 4‐DAG wild‐type and *IDD16*‐RNAi seedlings were determined by qRT‐PCR. Values are means ± SE from three biological replicates. (e–g) Compared to wild‐type Col (e), *IDD16*‐RNAi plants (f, g) showed increased stomatal density in abaxial epidermis of the cotyledons from 8‐DAG seedlings. Scale bars: 50 μm. (h) Stomata density obtained from the samples in (e–g). Three areas in the midleaf region were imaged per leaf. Data are the mean of six individual plants (mean ± SE,* n* = 6). Asterisks indicate statistical significance based on Student's *t* test; ** *P* < 0.01.

To determine the localization of IDD16 protein, a C‐terminus fusion of IDD16 with a green fluorescence protein (GFP) driven by the *IDD16* promoter (*IDD16pro:IDD16‐GFP*) was introduced into *Arabidopsis* plants. The IDD16 protein is expressed in epidermal cells only at very early stage of leaf development (Figures 5a and S4a). We analysed the level of IDD16 protein in adaxial epidermal cells of 1‐DAG cotyledons. In contrast to *SPCHpro:nucGFP*, which persisted in the stomatal lineage cells (MacAlister *et al*., 2007), IDD16 expression was absent in meristemoid cells and newly formed guard cells (Figure 5a). The specific cells expressing IDD16 might include newly formed pavement cells, and some un‐differential cells which could further enter the stomatal linage (Figure 5a). However, these two types of cells are very hard to tell apart, because we could only differentiate them according to morphology and cell size. As shown in Figure S4A, the expression pattern of IDD16 in 2‐DAG seedling cotyledons is consistent with Figure 5a. The IDD16 signal was more abundant in epidermis of young cotyledons, but not in the mesophyll cells (Figure S4c). The GFP fluorescence was also detected in the trichome of newly emerged true leaves (Figure S4B). No signal could be found in the root elongation zone (Figure S4D) or root tips (Figure S4E). This expression patterns suggested that IDD16 negatively regulated stomatal initiation at the early stage of leaf development. Increasing *IDD16* expression within its native domain (*IDD16pro:IDD16‐GFP*) in the wild‐type background also greatly inhibited the stomatal initiation (Figure 5b and c).

### Genetic interactions between *IDD16* and other stomatal regulators

TMM LRR receptor‐like protein (RLP) and *STOMATAL DENSITY AND DISTRIBUTION1* (*SDD1*) which encodes a subtilisin‐type proteinase, were identified as regulators of proper stomatal patterning (Berger and Altmann, 2000; Nadeau and Sack, 2002). TMM and SDD1 negatively regulate stomatal formation rather than promoting it; *tmm* and *sdd1* mutants make supernumerary stomata with abnormal spacing (Berger and Altmann, 2000; Nadeau and Sack, 2002). To examine whether *IDD16* overexpression can also reduce the stomatal initiation in these mutants, *IDD16* was overexpressed in *tmm* and *sdd1* under the control of the *35S* promoter. As shown in Figure S5, the stomatal density in these mutants was also reduced by the overexpression of *IDD16* in a dose‐dependent manner, indicating that the function of IDD16 in stomatal development was independent of TMM and SDD1.

### Stomatal densities were enhanced in *IDD16*‐RNAi plants

To characterize the phenotype of *idd16* mutants we obtained *idd16* mutants seeds from ABRC, but they were not germinated. We also try to knock out the *IDD16* gene using CRISPR/Cas9 gene‐editing technology, unfortunately, we did not get the null mutations (Brooks *et al*., 2014; Doudna and Charpentier, 2014). To assess whether *IDD16* is required for proper stomatal development, we silenced its expression using RNAi, designed from an *IDD16* transcript‐specific region. As shown in Figure 5d, *IDD16* transcript levels were specifically reduced in two independent RNAi lines, RNAi#2 and RNAi#3. The expression of two close homologs, *IDD14* and *IDD15*, was not affected in RNAi lines (Figure S6). Subsequently, we examined the expression of *SPCH* and other stomatal development genes in *IDD16*‐RNAi lines. As shown in Figure S7, the expression level of these genes was enhanced to some extent. As expected, the stomata density of both RNAi lines was enhanced as compared to wild‐type plants (Figure 5e‐h). These results suggested that the wild‐type level of *IDD16* expression was required for proper stomatal patterning and that *IDD16* is an intrinsic regulator of stomatal development in *Arabidopsis*.

### IDD16 directly binds to the *SPCH* promoter

**Figure 6:**
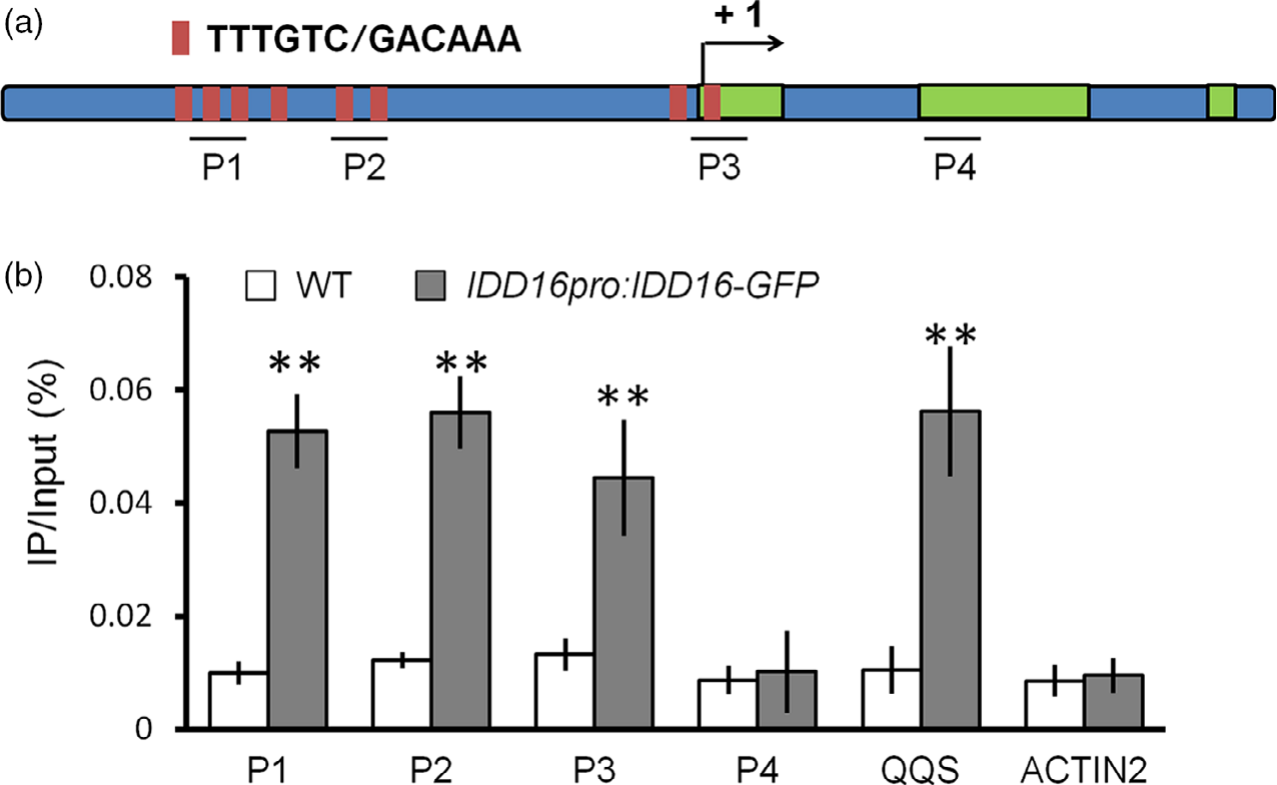
IDD16 directly associates with the *SPCH* promoter. (a) *SPCH* locus comprising the 2‐kb promoter region and transcribed region. Black lines denote fragments amplified in ChIP‐qPCR (b). Red bar represents the core binding sites (TTTGTC or GACAAA) of IDD proteins. (b) qPCR of fragments (as in a) from ChIP of *IDD16pro:IDD16*‐*GFP* seedlings and WT with anti‐GFP antibody. Negative control P4 for ChIP‐qPCR are located in the second exon. Values are means ± SE from three biological replicates. Asterisks indicate statistical significance based on Student's *t* test; ** *P* < 0.01.

The results described above indicated that IDD16 could negatively affect *SPCH* promoter activity (Figure 4a‐c). Therefore, we assessed whether IDD16, as a transcription factor, could regulate *SPCH* transcription by directly binding to the *SPCH* promoter region. To this end, we first performed an *in silico* analysis of the *SPCH* promoter for the presence of a 11‐bp cis‐element (T‐T‐T‐G‐T‐C‐G/C‐T/C‐T/A‐T/A‐T), previously reported as a binding site for IDD transcription factors (Seo *et al*., 2011). Eight ‘TTTGTC’ elements, identical to the core sequence of the 11‐bp binding site, were found both in the promoter and the first exon of *SPCH* (Figure 6a). Next, the Maximized Objects for Better Enrichment (MOBE)‐ChIP assays were performed to determine whether IDD16 could bind to the *SPCH* promoter *in vivo*. DNA samples from *IDD16pro:IDD16‐GFP* was immuno‐precipitated with anti‐GFP antibody followed by qPCR to amplify the promoter fragments relative to that from wild‐type. The results demonstrated that the P1, P2 and P3 regions of the *SPCH* promoter comprised conserved ‘TTTGTC’ sequences, but not the P4 region, were significantly enriched in the *IDD16pro:IDD16‐GFP* sample (Figure 6b). As a positive control, the promoter fragment from *QUA‐QUINE STARCH (QQS)* gene, a known target of IDD transcription factors, was also enriched (Cui *et al*., 2013; Seo *et al*., 2011). Taken together, these results suggested that IDD16 is a direct regulator for *SPCH* transcription. As shown in Figure S8, we analysed the occurrence of the IDD binding sites in the promoters of the *SPCH* homologous genes in crop species. IDD binding sites ‘TTTGTC’ can be found in the promoters of *SPCH* genes in both monocots and dicots. For example, two IDD binding sites were found in *SPCH* promoter from *Ananas comosus*,* Brachypodium distachyon* and *Solanum lycopersicum*. Four to seven IDD binding sites were found in the promoter and the first exon of *SPCH* in *Zea mays*,* Ricinus communis*,* Brassica oleracea capitata*,* Brassica rapa*,* Glycine max* and *Medicago truncatula*. This result suggested that the regulatory module of IDD16 in stomatal development may be conserved across angiosperm species.

### Constitutive overexpression of *IDD16* increased water‐deficit tolerance

**Figure 7:**
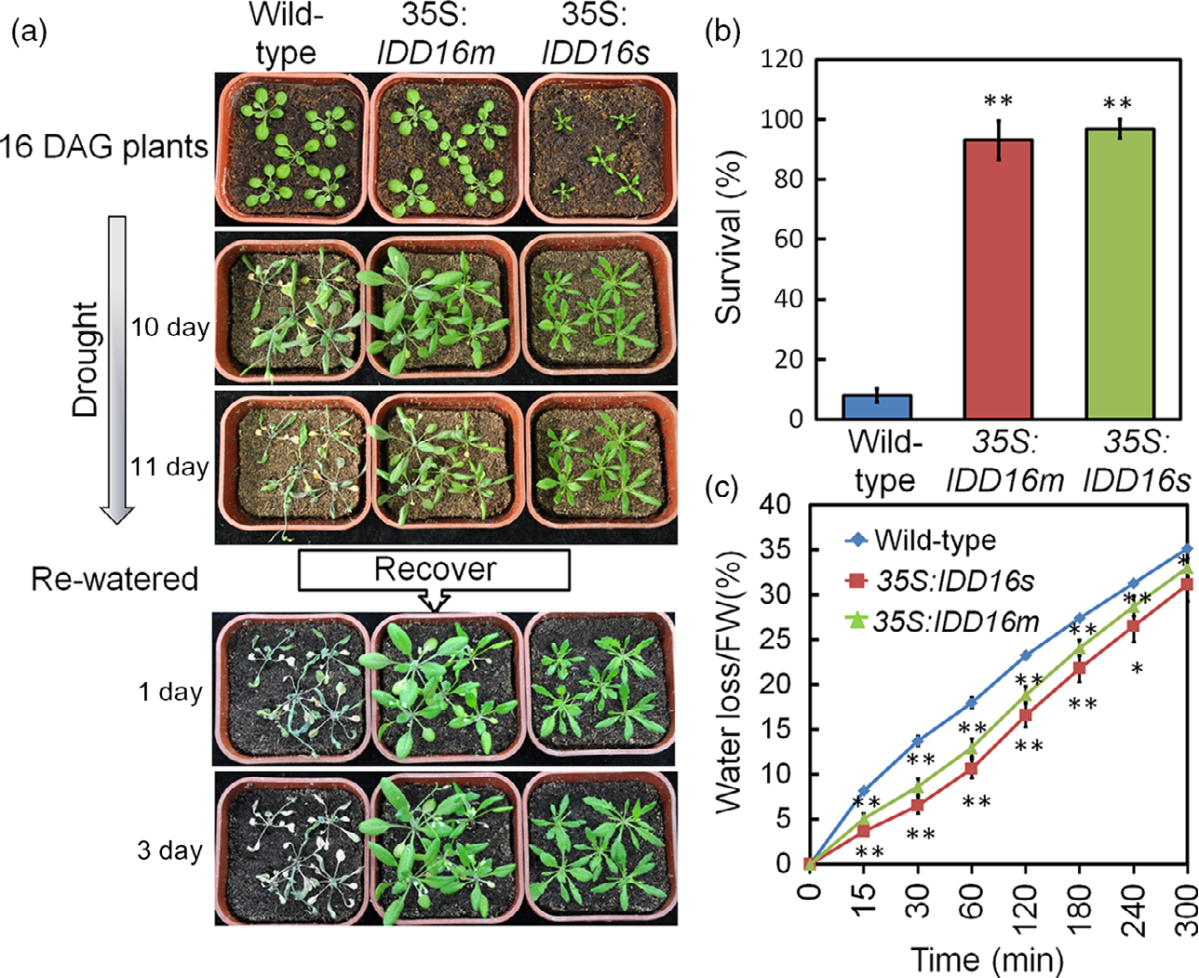
*IDD16*‐OE plants exhibit enhanced water‐deficit stress tolerance that correlates with reduced water loss. (a) 16‐DAG well‐watered wild‐type, 35S: *IDD16m* and 35S: *IDD16s* plants (top panel) were withdrawn from irrigation for 11 days and then fully re‐watered. Pictures were taken at the indicated times. Experiments were repeated three times with the similar results. More than 60 plantlets for each genotype were used in each experiment. (b) Seedlings survival rates were determined 3 days after re‐watering. Data are from three independent experiments and shown as mean ± SE. (c) Tissue water loss. Aerial parts of 3‐week‐old well‐watered plants were detached, weighed immediately (0 min), allowed to dry under ambient conditions and weighed at the indicated times. Water loss was calculated as the percentage of the fresh weight at time zero. Values are means ± SE from four biological replicates. Asterisks indicate statistical significance based on Student's *t* test; ** *P* < 0.01, * *P* < 0.05.

Stomata accounts for the bulk of the leaf water transpiration. Given the role of IDD16 in regulating stomata development (Figures 2 and 3) and the effects of stomatal density on plant water use efficiency (WUE) (Meng and Yao, 2015), we evaluated the tolerance of the *IDD16‐*OE plants to severe drought stress. 35S:*IDD16s*, 35S:*IDD16m* and wild‐type plants were grown in separate pots with equal amounts of soil and water and the stress treatment was applied at 16 days after germination (DAG) by withdrawing watering (Figure 7a). After re‐watering, following 11 days of water‐deficit stress, the growth of wild‐type plants was severely inhibited while the *IDD16*‐OE lines displayed better re‐growing. After 3 days of re‐watering the survival rate was 93% and 97% for 35S:*IDD16m* and 35S:*IDD16s,* respectively and only 8% for wild‐type plants (Figure 7b), indicating a significant drought tolerance of the transgenic plants. The drought tolerance of the *IDD16*‐OE plants were further supported by the better ability of the *IDD16*‐OE (35S:*IDD16s* > 35S:*IDD16m *> wild‐type) leaves to withhold water as shown by their reduced leaf water loss (Figure 7c).

The reduced leaf water loss in 35S:*IDD16m* plants may be caused by altered stomatal aperture or stomatal density. We then also compared stomatal apertures under treatment with different concentrations of ABA. The first leaves of 35S:*IDD16m* and wild‐type were incubated in a buffer under strong light conditions for 6 h to have stomata fully open. Then, the leaves were treated with different concentrations of ABA for 2 h. No significant difference in leaf abaxial stomatal aperture was observed between 35S:*IDD16m* and wild‐type plants with or without ABA treatment (Figure S9A and B), suggesting that *IDD16* regulates stomatal density but not stomatal opening/closing.

### *IDD16*‐OE Plants have reduced transpiration and improved integrated WUE

**Figure 8:**
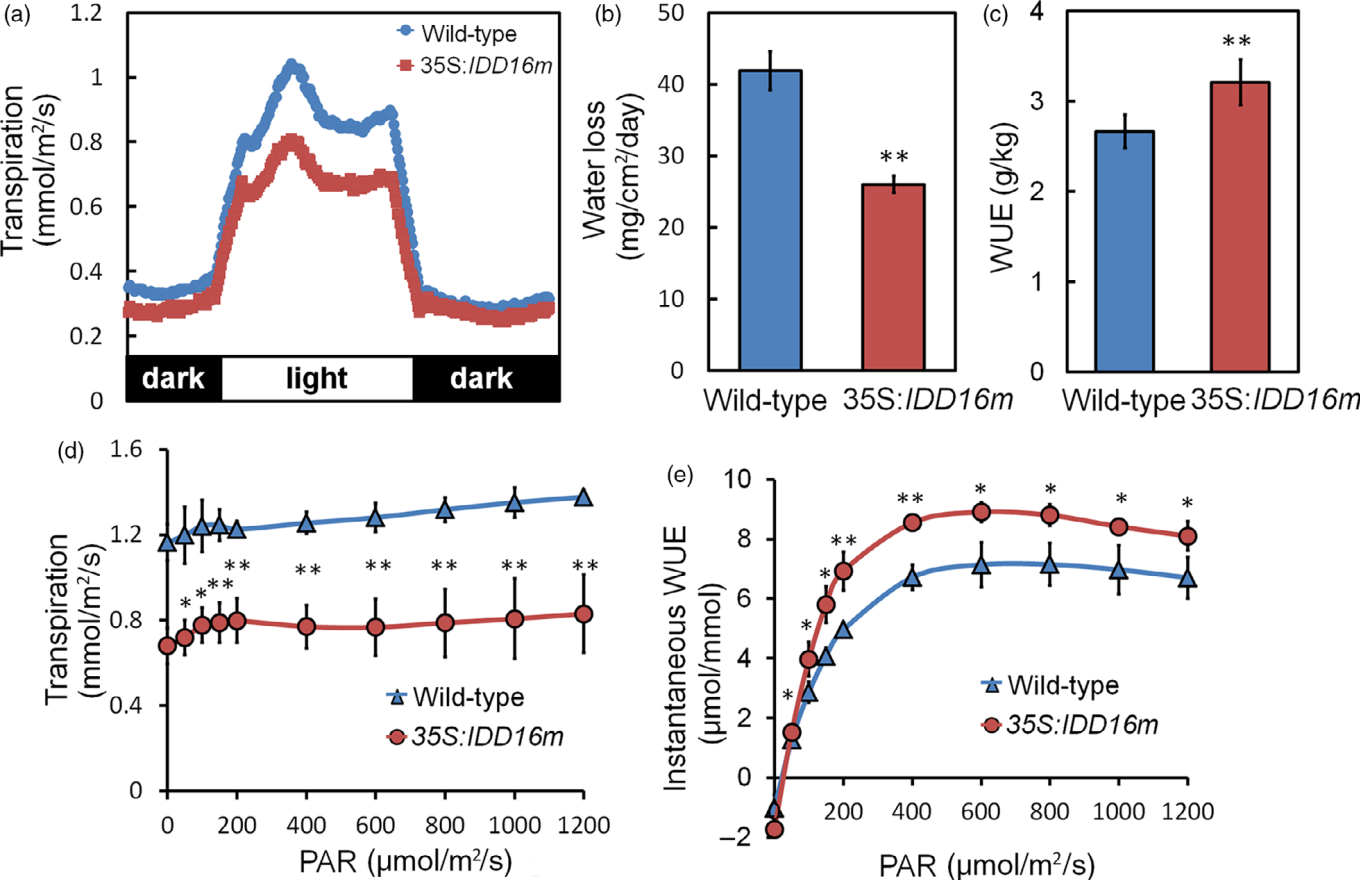
Reduced transpiration and improved integrated WUE in *IDD16*‐OE plants. (a) Diurnal transpiration rate and (b) light period water loss of 5‐week‐old wild‐type and 35S: *IDD16m* plants grown under a 12‐h‐light/12‐h‐dark photoperiod were determined by gravimetric analysis. Values are means ± SE from four biological replicates, with each replicate include more than 12 plantlets. (c) Water use efficiency (WUE) as determined by the sum of daily transpiration and biomass production over 7 days. Plants were grown under a light intensity of PAR = 120 μmol/m^2^/s. Values are means ± SE from three biological replicates, with each replicate include more than 20 plantlets. (d) Leaf transpiration and (e) instantaneous WUE were determined on individual leaves of 5‐week‐old wild‐type (Col‐0) and 35S: *IDD16m* plants using a Li‐Cor 6400 gas exchange system, values are the Mean ± SE (*n* = 4). Asterisks indicate statistical significance based on Student's *t* test; ** *P* < 0.01, * *P* < 0.05.

To understand the physiological mechanisms underlying the water‐deficit stress tolerance and reduced water loss of *IDD16*‐OE plants, transpiration rates of 35S:*IDD16m* were measured by gravimetric analyses over diurnal light/dark periods. 35S:*IDD16m* plants were used because of their relatively normal leaf shape and size (Figure 1a). 35S:*IDD16m* plants displayed lower transpiration rates than wild‐type plants during the light period (Figure 8a), resulting in a reduced daily water loss (Figure 8b). The significant reduction in transpiration (Figure 8a and b) was not associated with decreased shoot dry weight (Figure S10A) in 35S:*IDD16m*. Consequently, 35S:*IDD16m* had higher integrated WUE (biomass/water use) than the wild‐type (Figure 8c).

The similar dry weight of 35S:*IDD16m* and wild‐type plants suggested that CO_2_ assimilation in the transgenic plants might not be reduced to the same extent as the transpiration rates under our experimental conditions. Gas exchange (water and CO_2_) of fully expanded leaves from wild‐type and 35S:*IDD16m* was determined using an infrared gas analyser. At low light intensity levels (PAR < 200 μmol/m^2^/s), 35S:*IDD16m* leaf transpiration rates were 37% lower than that of wild‐type plants (Figure 8d), while the net CO_2_ assimilation rates were not significantly different from that of wild‐type (Figure S10B). As a result, 35S:*IDD16m* plants had higher instantaneous WUE (CO_2_ assimilation/transpiration; Figure 8e). Internal CO_2_ concentration (Ci) in leaves of 35S:*IDD16m* was lower than that in wild‐type leaves (Figure S10C), possibly due to a reduced CO_2_ flux from the air to the sub‐stomatal cavity in the transgenic plants.

## Discussion

In this report, we provided evidences showing that IDD16 plays an important role in the regulation of stomatal development via its binding to the *SPCH* promoter to repress its transcription. In *Arabidopsis*, SPCH is required for the initiation and proliferation of stomatal lineage cells. SPCH‐driven asymmetric and self‐renewing divisions allow flexibility in stomatal production (Lau *et al*., 2014). However, which transcription factor tightly controls the SPCH‐mediated stomatal initiation and proliferation is unclear. Here, we showed that IDD16 functions as a negative regulator of *SPCH* transcription to control the stomatal initiation (Figures 2, 3, 4, 5). This conclusion was supported by the following: (i) *SPCH* transcript levels and the *SPCH:nucGFP* signal in the abaxial epidermis was significantly reduced in *35S:IDD16* plants; (ii) ChIP‐qPCR analysis showed that IDD16 protein could directly interact with the *SPCH* promoter, indicating that *SPCH* is a direct downstream target of IDD16.

SPCH has been shown to be negatively regulated through phosphorylation by MAPKs and GSKs families (Gudesblat *et al*., 2012; Lampard *et al*., 2009). However, *MAPKs*, GSKs and *CDKs* upstream of *SPCH* are all broadly expressed in the leaf, so there must be other regulators expressing in specific cells to produce the normal pattern of SPCH activity. Here, we showed that IDD16, as a key transcription factor repressing the expression of *SPCH*, is a putative negative regulator of *SPCH* at the transcriptional level. Our data showed that the decrease in stomatal density in *IDD16‐OE* plants was correlated with a reduced number of stomatal precursor cells, such as MMC and meristemoid (Figures 2i‐q and 3g‐o). However, the further development of these precursor cells into GMC appeared not affected, suggesting that *IDD16* overexpression specifically impaired the transition of protodermal cells into stomatal lineage cells. To date, the molecular mechanism of MMC generation is not very well understood. *SPCH* is an early regulator of this process, since no MMC formation could be observed in *spch* mutant (MacAlister *et al*., 2007), thus suggesting that the observed inhibition of stomatal initiation displayed in *IDD16‐OE* plants was due to reduced *SPCH* expression (Figure 2). With regards to the other eight down‐regulated genes (Figure 4a), since all of them are specifically expressed in stomatal precursor cells, such as meristemoid mother cells, meristemoid cells, guard mother cells or newly formed stomata, the reduced expression of these eight genes can be logically attributed to suppressed formation of stomatal precursor cells in *IDD16‐OE* plants (Figures 2i–q and 3g–o). For example, the reduced *FAMA* transcript levels were due primary to a lesser amount of cells expressing *FAMA*, rather than the inhibition of *FAMA* transcription activity (Figure S3).

We also showed that the inhibitory effect of IDD16 on stomatal initiation was different between abaxial and adaxial epidermis (Figures 2 and 3). In *IDD16‐OE* plants, stomatal initiation on abaxial epidermis was more dramatically affected than on the adaxial side. In some of the transgenic plants highly expressing *IDD16*, no stomata could be found on the abaxial epidermis (Figure 2b–h) while considerable stomata were still present on the adaxial side (Figure 3a–f), allowing the transgenic plants to complete their life cycle. Many known regulators of stomatal development, such as *SPCH*,* TMM* and *SDD1*, usually exert similar effects on both abaxial and adaxial epidermis. For example, homozygous *SPCH* mutation inhibited stomatal development equally on both abaxial and adaxial epidermis (MacAlister *et al*., 2007). Both *tmm* and *sdd1* mutants showed similar increase in stomatal density on both epidermis (Berger and Altmann, 2000; Nadeau and Sack, 2002). The acquisition and maintenance of adaxial‐abaxial leaf polarity is controlled by a complex, redundant gene regulatory network comprised of several highly conserved transcription factor families (Husbands *et al*., 2009; Moon and Hake, 2011). The adaxial‐abaxial leaf polarity eventually leads to the asymmetric distribution of cell types in the mature leaf (Moon and Hake, 2011). For example, in *Arabidopsis*, both the shape and the patterning of pavement cells are different between abaxial and adaxial epidermis. Similarly, there are also some difference in stomatal density and patterning on abaxial and adaxial epidermis (Figures 2b and 3a), indicating that the initiation and proliferation of stomatal lineage cells is not equally regulated. Our results showed that IDD16 may be controlling stomatal initiation and proliferation on abaxial and adaxial epidermis differentially. How developmental signals are integrated by IDD16 to optimize these processes requires further investigation.

In summary, our research shows that IDD16 acts as a negative regulator of stomatal initiation by directly repressing the expression of *SPCH*, which advances our understanding of the regulation of stomatal development. Notably, the stomatal initiation in *IDD16*‐OE plants was reduced by the overexpression of *IDD16* in a dose‐dependent manner. This provided us with a useful tool to manipulate stomatal density in transgenic plants by selecting a proper expression level of *IDD16* transgene. We showed that the overexpression of *IDD16* reduced the stomatal density in the leaf epidermis (Figure 2), enhancing plant WUE and improving the plant tolerance to severe drought stress (Figures 7 and 8). Not only our results shed light on the transcriptional regulation of *SPCH*, but also provide a practical tool for increasing plant WUE through the manipulation of *IDD16* expression.

## Experimental procedures

### Plant materials and growth conditions

All *Arabidopsis thaliana* plants used in this study are in the Col‐0 (ecotype Columbia) background. *SPCHpro:nucGFP* and *FAMA:GFP* have been described previously (MacAlister *et al*., 2007; Ohashi‐Ito and Bergmann, 2006). The loss‐of‐function mutants *tmm‐1* (Nadeau and Sack, 2002) and *sdd1* (Berger and Altmann, 2000) were used. Transgenic plants and mutants were sown on solid MS medium and stratified at 4 °C for 3 days in dark, before being transferred to an incubator for germination at 22 °C with a 16‐h light/8‐h dark photoperiod (light intensity of 120 μmol/m^2^/s).

### Plasmid constructions and plant transformation

Plant binary vectors based on Gateway cloning technology (Invitrogen) were used for most constructions. cDNA of *IDD16* was inserted into pENTR/TOPO and recombined into the destination vectors pMDC32 or pMDC85. A translational fusion for IDD16 was made by replacing the 2 × 35S with the 2.6 kb promoter *of IDD16* in pMDC85. DNA fragments for *IDD16* silencing by dsRNA were PCR‐amplified from cDNA and inserted into pDONR221 and this vector was recombined into pK7GWIWG2. Plants were stably transformed using *Agrobacterium tumefaciens*‐mediated transformation (strain C58) using standard protocols (Clough and Bent, 1998). Transgenic lines were selected on 1/2; MS medium containing 30 mg/L hygromycin or 60 mg/L kanamycin.

### Physiological analyses

Severe drought stress was induced by withholding water from plants (16 DAG) grown in soil (Pindstrup Mosebrug, 16093421/LV/SEEDING; pH 5.0; 0–10 mm; 300 L; dry weight, 34 ± 0.1 g) in a growth room (16‐h light/8‐h dark, 22°C). Watering was withheld for 11 days, plants were re‐watered and the plant survival rates were assessed 3 days later.

To measure water loss in detached leaves, the leaves were removed from 3‐week‐old wild‐type and transgenic plants were grown under normal growth conditions. The detached leaves were placed on a laboratory bench at ambient temperature and weighed periodically. Water loss rates were calculated as [(initial fresh weight–final fresh weight)/initial fresh weight] × 100.

Stomatal closing assays were conducted as described by Ren *et al*. (2010). The first leaves were floated in solutions containing 10 mm KCl, 10 mm Mes‐Tris, pH 6.2, and exposed to light (150 μmol/m^2^/s) for 6 h. ABA was added to the solution at 1 and 10 μm to assay for stomatal closing. After ABA treatment for 2 h, images of abaxial epidermis were captured at 500× magnification using an Olympus BX51 microscope. Stomatal apertures were measured using the ImageJ program (National Institutes of Health).

Whole‐plant transpiration of 5‐week‐old plants was determined by a gravimetric method as described previously (Yoo *et al*., 2010). Individual plants were grown in 200‐mL containers with a 12‐h light/12‐h dark photoperiod (light intensity of 120 μmol/m^2^/s). During the period that transpiration was measured, each container was covered with a polyethylene wrap to prevent water evaporation from the soil surface. Plants were placed onto a balance and the weight of each container was determined every 5 min for a period of 72 h. At the end of the experiment, total leaf area was determined from photographs of excised leaves using the ImageJ program (National Institutes of Health). Transpiration rates (mmol water/m^2^/s) were calculated based on gravimetric water loss rates and leaf area data.

Integrated WUE was calculated as the final shoot dry weight divided by the total water loss over a period of 1 week of plants grown in soil of 200‐mL containers (Tanaka *et al*., 2013). Shoot biomass was determined by harvesting two groups of plants at 35 and 42 DAG. Between the first and second harvests, the soil surface was covered with polyethylene to prevent water evaporation. Transpiration for the second group of plants was determined every day by the gravimetric method throughout 7 consecutive  days. WUE was calculated from the dry matter accumulation and cumulative transpiration between the first and second harvest.

### Photosynthetic measurements

Mature leaves of 5‐week‐old plants were used for measuring photosynthesis, assessing leaf gas exchange of fully expanded leaves, (transpiration, stomatal conductance, and net CO_2_ assimilation) using a LI6400XT infrared gas analyser (LI‐COR Biosciences). Plants were grown in soil for 35 days in 400‐mL containers under short‐day conditions (12 h/12 h light/dark). Gas exchange was measured at PAR levels of 1200, 1000, 800, 600, 400, 200, 150, 100, 50, 25 and 0 mmol/m^2^/s. Instantaneous WUE was calculated as the net CO_2_ assimilation rates divided by the transpiration rates.

### Stomatal phenotyping

Stomatal density (stomata number per area), stomatal index (ratio of stomata to total epidermal cells, including stomata, stomatal precursor cells, and pavement cells), the number of stomatal precursor cells, and pavement cell density (pavement cells per area) were obtained from a leaf area of 0.2 mm^2^ in the midleaf region. Only the eighth rosette leaf or cotyledons were used for analysis and three areas in the midleaf region, between the midvein and leaf edge, were imaged per leaf. In all calculations, stomata were considered to be a pair of guard cells. Stomatal precursor cells were identified as cells at the meristemoid or GMC stage.

### RNA isolation and quantitative RT‐PCR

Total RNAs were isolated using RNAprep pure Plant kit (TIANGEN). cDNA was synthesized from 2 μg of total RNA with oligo(dT)_18_ and Superscript™ reverse transcriptase (Invitrogen). The reverse transcription mix was diluted 10 times and 1 μL was used for quantitative real‐time PCR, performed with the Stratagene MX3000P system (Agilent Stratagene, Santa Clara, CA) and KAPA SYBR^@^ FAST Master Mix (KK4601; KAPABIOSYSTEMS). Quantification was performed using the 2^−ΔΔCt^ method (Livak and Schmittgen, 2001), where ΔΔCt is the difference in the threshold cycles between specific genes and the reference housekeeping genes, which were *eIF4A* and *ACT7* for expression analyses or input DNA for ChIP assays. The sequences of all the gene‐specific primers are listed in Table S1.

### Microscopy

To obtain differential interference contrast (DIC) images, cotyledons or leaves were treated with destaining solution (containing 75% ethanol and 25% acetic acid) for 30 min or overnight at room temperature until the chlorophyll was cleared. After a treatment with basic solution (7% NaOH in 60% ethanol) for 3 h at room temperature, the samples were rehydrated via an ethanol series (40%, 20%, and 10%) for 15 min at each step. Two images at 200× magnification (0.2 mm^2^) were captured per cotyledon using an Olympus BX51 microscope.

For multicolor images of GFP and propidium iodide (PI), transgenic seedlings were observed on Leica TCS SP5. Cell outlines were visualized by staining with 0.5% propidium iodide (PI) for 1–2 min. The 488 nm laser was used to excite GFP and the 543 nm laser was used to excite PI. The emission filter was 500–515 nm for GFP and 591–635 nm for PI. The Z‐stack projection images were taken at the interval of 1 μm, covering the thickness of the entire cell. All microscope control settings, such as those for pinholes, gain, detector range and microscope objectives, were identical for all the unmixing controls. In order to compare the intensity of GFP signal, the focus was adjusted to best visualize the GFP signal.

### MOBE‐ChIP assays

MOBE‐ChIP was performed with 24 g (fresh weight) of 4‐DAG seedlings of *IDD16pro:IDD16‐GFP* transgenic lines and WT. Anti‐GFP antibody was used to pull down the chromatin, as described previously (Lau and Bergmann, 2015). IP/input (%) was calculated by comparing the Ct values of immunoprecipitate and input from each genotype.

### Statistical analysis

Comparisons between two groups were made using Student's *t* test. The values of control conditions or WTs were considered as references.

### Accession numbers

The gene sequences mentioned in this study can be found in the Arabidopsis Genome Initiative database under the following accession numbers: AT1G25250 (*IDD16*), AT5G53210 (*SPCH*), AT3G13920 (*eIF4a*), AT3G06120 (*MUTE*), AT3G24140 (*FAMA*), AT1G80080 (*TMM*), AT1G04110 (*SDD1*), AT2G20875 (*EPF1*), AT1G34245 (*EPF2*), At5g60880 (*BASL*), At4g31805 (*POLAR*), AT3G26744 (*ICE1*), AT1G12860 (*ICE2*), AT1G63700 (*YDA*), AT4G12970 (*STOMAGEN*), AT2G26330 (*ERECTA*), At5g62230 (*ERL1*), AT5G07180 (*ERL2*), AT3G12280 (AT3G12280), AT1G04710 (*PKT4*), AT3G48750 (*CDKA1*), AT3G45640 (*MPK3*), AT1G51660 (*MKK4*), At3g18780 (ACTIN2), AT3G30720 (*QQS*).

## Author contributions

S.L.Q. and X.J.H. designed the experiments; S.L.Q. and Q.F.L. performed experimental work; S.L.Q., Q.F.L., X.J.F., H.L.H., J.L., L.Z. and S.W. performed data analysis; E.B. and J.L. helped with a critical discussion and experimental materials related to the work; S.L.Q. and X.J.H. wrote the manuscript with great help from E.B.

## Conflict of interest

The authors declare no conflict of interest.

## Supporting information


**Figure S1** Correlation between stomatal density in cotyledons and *IDD16* transcripts level.
**Figure S2** Analysis of the expression of the genes related to stomatal development.
**Figure S3** The effect of *IDD16* overexpression on *FAMA* expression.
**Figure S4** The expression pattern of IDD16 protein.
**Figure S5** Effect of *IDD16* overexpression on stomatal density in different genetic backgrounds.
**Figure S6** The expression of *IDD14* and *IDD15* were not affected in *IDD16*‐RNAi lines.
**Figure S7** The expression of genes related to stomatal development was enhanced in *IDD16*‐RNAi seedlings.
**Figure S8** The core binding sites of IDD proteins in promoter of *SPCH*‐like genes from angiosperms.
**Figure S9** 35S:*IDD16m* plants exhibited similar stomatal aperture in response to ABA treatment compared with wild‐type plants.
**Figure S10** Growth and carbon assimilation of 35S:*IDD16m* plants were not affected under normal growth condition.
**Table S1** Primers used in this study.
