# Supplementary material for: IDD16 negatively regulates stomatal initiation via trans‐repression of SPCH in Arabidopsis
Source: Plant Biotechnol J. 2019 Mar 27;17(7):1446–57. doi: 10.1111/pbi.13070 (PMC6576023; doi:10.1111/pbi.13070)
Supplement: Supplementary file 1 — Figure S1 Correlation between stomatal density in cotyledons and IDD16 transcripts level. Figure S2 Analysis of the expression of the genes related to stomatal development. Figure S3 The effect of IDD16 overexpression on FAMA expression. Figure S4 The expression pattern of IDD16 protein. Figure S5 Effect of IDD16 overexpression on stomatal density in different genetic backgrounds. Figure S6 The expression of IDD14 and IDD15 were not affected in IDD16‐RNAi lines. Figure S7 The expression of genes related to stomatal development was enhanced in IDD16‐RNAi seedlings. Figure S8 The core binding sites of IDD proteins in promoter of SPCH‐like genes from angiosperms. Figure S9 35S:IDD16m plants exhibited similar stomatal aperture in response to ABA treatment compared with wild‐type plants. Figure S10 Growth and carbon assimilation of 35S:IDD16m plants were not affected under normal growth condition. Table S1 Primers used in this study. [file PBI-17-1446-s001.pdf]

Supporting Information

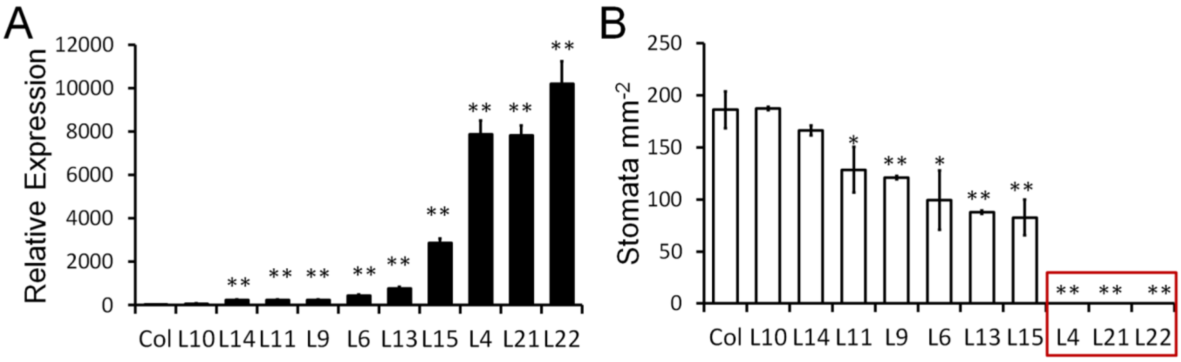

Supplemental Figure 1. Correlation between stomatal density in cotyledons and *IDD16* transcripts level.

(A) Expression level of *IDD16* in transgenic plants. qRT-PCR was performed on total RNA from 4-DAG wild-type and *IDD16*-OE plants. The expression level in wild-type was normalized as 1. **(B)** Stomatal density in cotyledons of 8-DAG *IDD16*-overexpression plants. Values are means ± SE from three biological replicates. Asterisks indicate statistical significance based on Student's t test; ** P < 0.01, * P < 0.05.

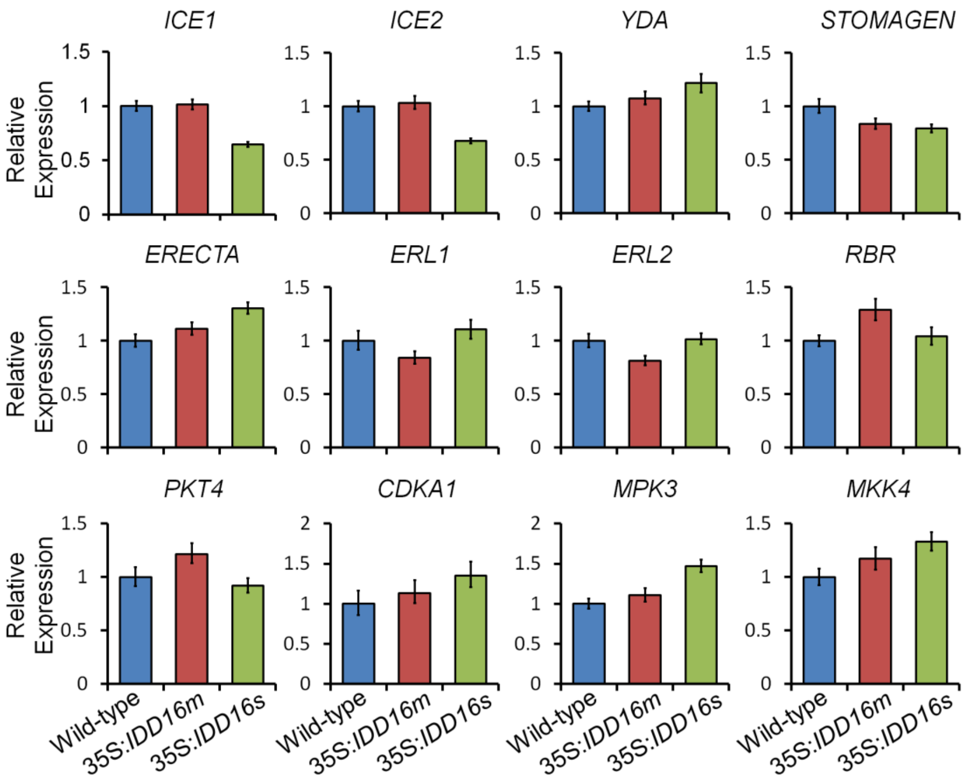

Supplemental Figure 2 Analysis of the expression of the genes related to stomatal development.

qRT-PCR was performed on total RNA from 4-DAG Wild-type and *IDD16*-OE plants. The expression level in wild-type was normalized as 1. Data represent means ± SE of three biological replicates.

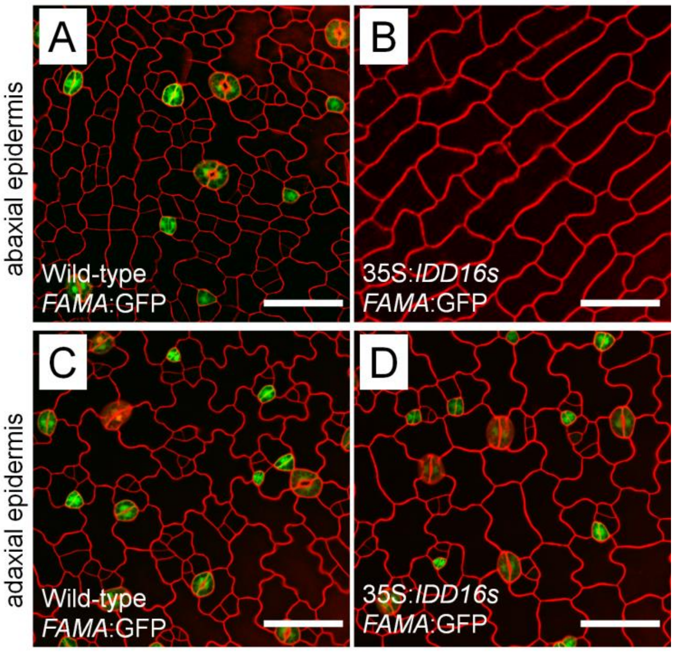

Supplemental Figure 3 The effect of *IDD16* overexpression on *FAMA* expression. (A and B) Confocal images of *FAMA*:GFP abaxial cotyledons in 2-DAG wild-type and *35S:IDD16s* background, respectively. **(C and D)** show the images of *FAMA*:GFP on adaxial epidermis of cotyledons. Epidermal cell periphery is highlighted by propidium iodide (red) staining. Scale bars: 50 μm.

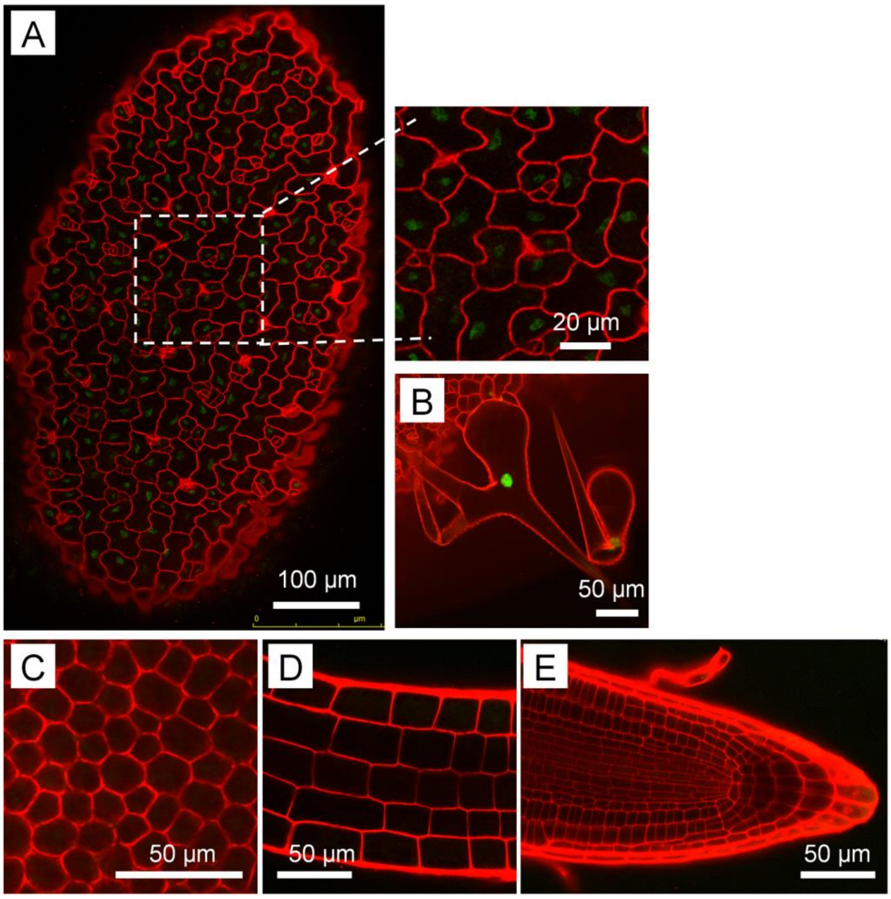

Supplemental Figure 4 The expression pattern of *IDD16* protein.

The expression of IDD16 protein was visualized in *IDD16pro:IDD16-GFP* transgenic lines under confocal microscopy. (A) The expression pattern of *IDD16* protein in adaxial epidermis of 2-DAG seedling cotyledons, GFP signal was absent in meristemoid cells and young guard cells. (B-E) The expression is strong in trichome of young leaves of 7 DAG seedlings (B), but almost undetectable in mesophyll cells of cotyledons (C), root elongation zone (D) and root tip (E) of 1-DAG seedlings. Cell periphery is highlighted by propidium iodide (red) staining.

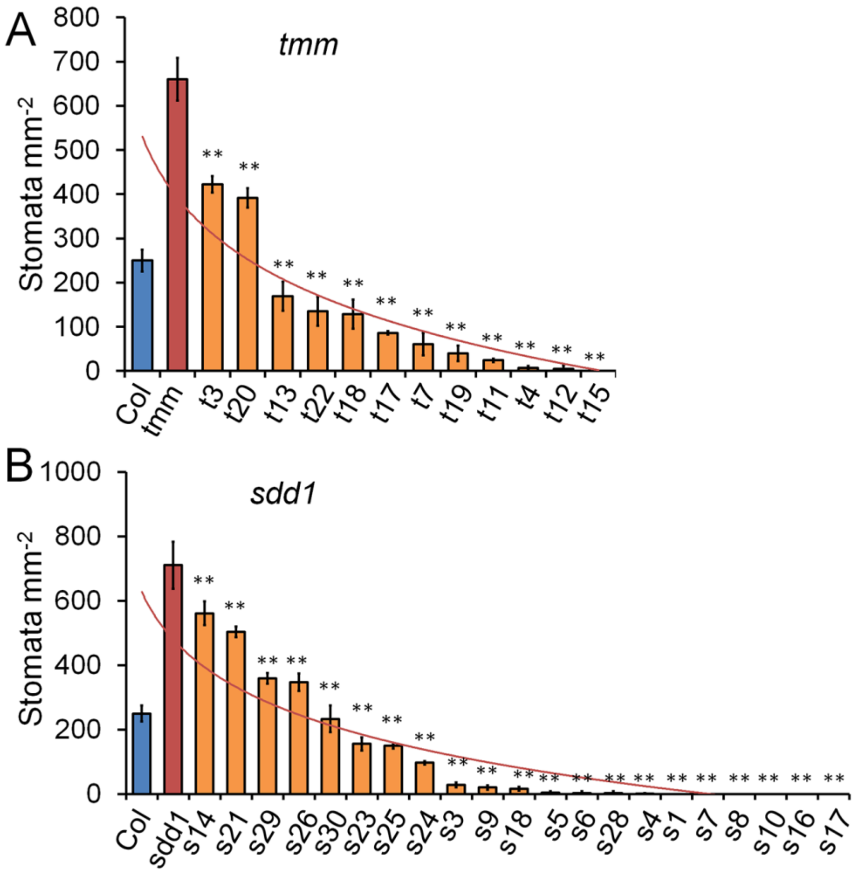

Supplemental Figure 5 Effect of *IDD16* overexpression on stomatal density in different genetic backgrounds.

Stomatal densities in abaxial epidermis of the cotyledons from 11-day-old *tmm* **(A)** and *sdd1* **(B)** seedlings containing *35Spro:IDD16* were analyzed. Values are means ± SE from three biological replicates. Asterisks indicate statistical significance from the background (*tmm* or *sdd1*) based on Student's t test; ** $P < 0.01$.

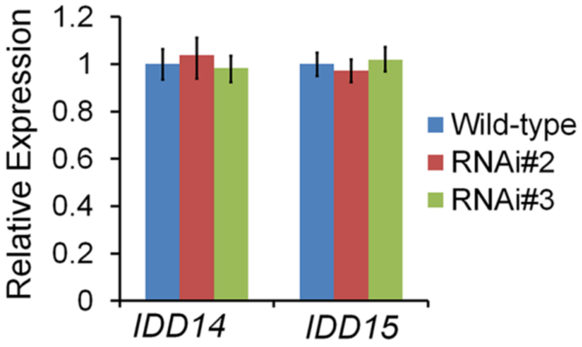

Supplemental Figure 6 The expression of *IDD14* and *IDD15* were not affected in *IDD16*-RNAi lines.

The expression levels of *IDD14* and *IDD15* in 4-DAG wild-type and *IDD16*-RNAi seedlings were determined by qRT-PCR. Values are means ± SE from three biological replicates. Primers are listed in Supplementary Table S1.

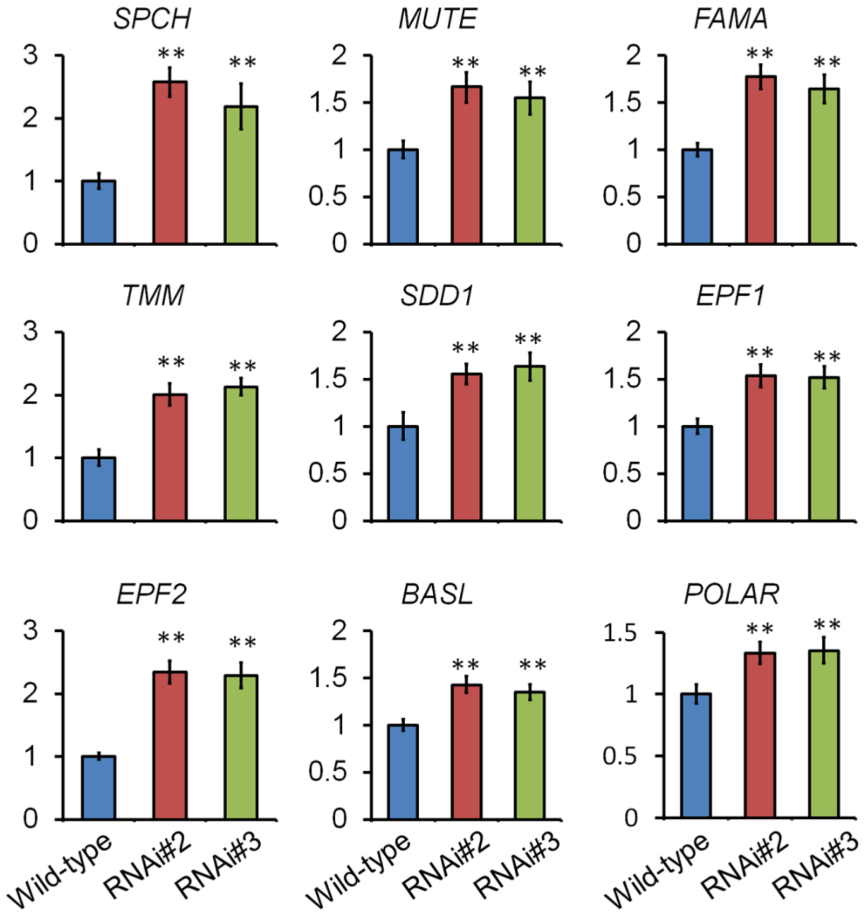

Supplemental Figure 7 The expression of genes related to stomatal development was enhanced in *IDD16*-RNAi seedlings.

qRT-PCR was performed on total RNA from 4-DAG wild-type and *IDD16*-RNAi seedlings. Values are means ± SE from three biological replicates. Asterisks indicate statistical significance based on Student's t test; ** $P < 0.01$.

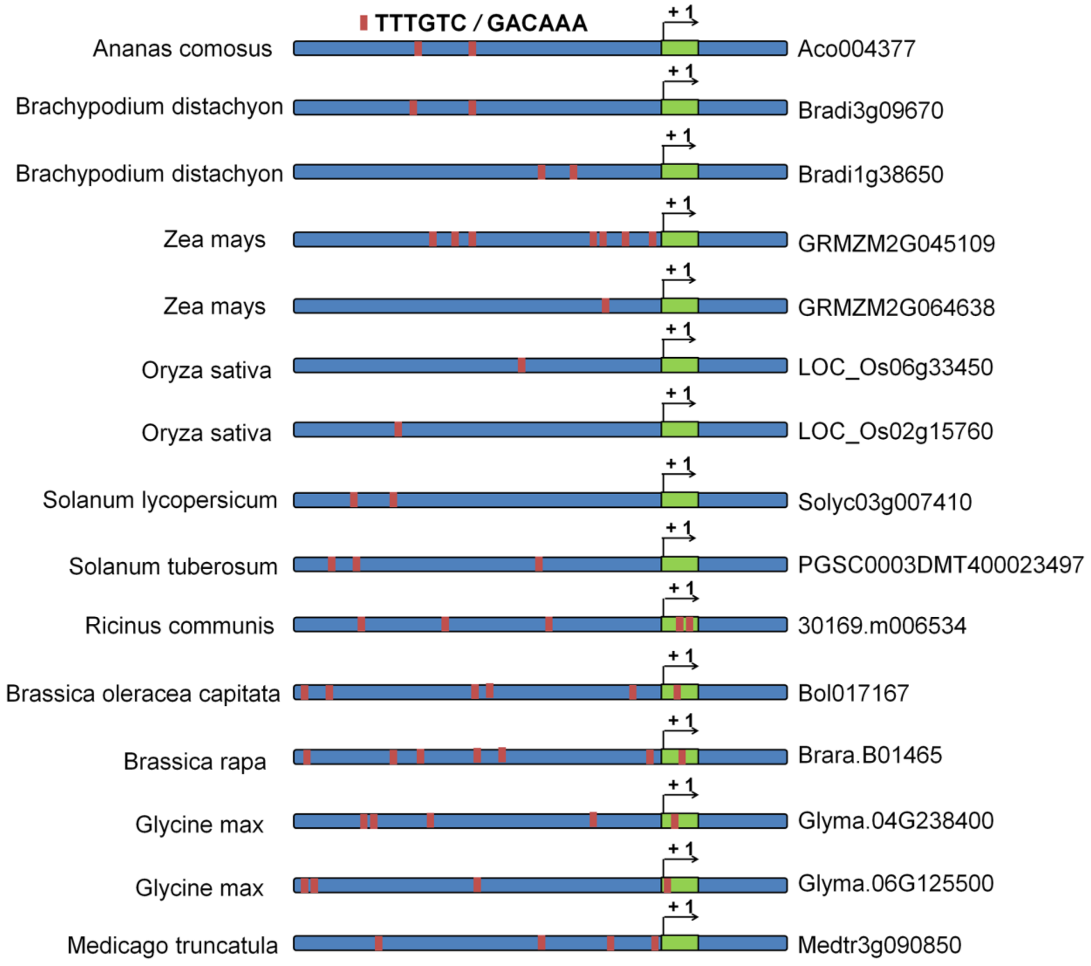

Supplemental Figure 8 The core binding sites of IDD proteins in promoter of *SPCH*-like genes from angiosperms.

The angiosperm genomes were searched through Phytozome v12 (https://phytozome.jgi.doe.gov/pz/portal.html#) by similarity search with the *SPCH* of *Arabidopsis*. 3-kb promoter region upstream of the initiation codon and the first exon were analyzed. Red bar represents the core binding sites (TTTGTC or GACAAA) of IDD proteins.

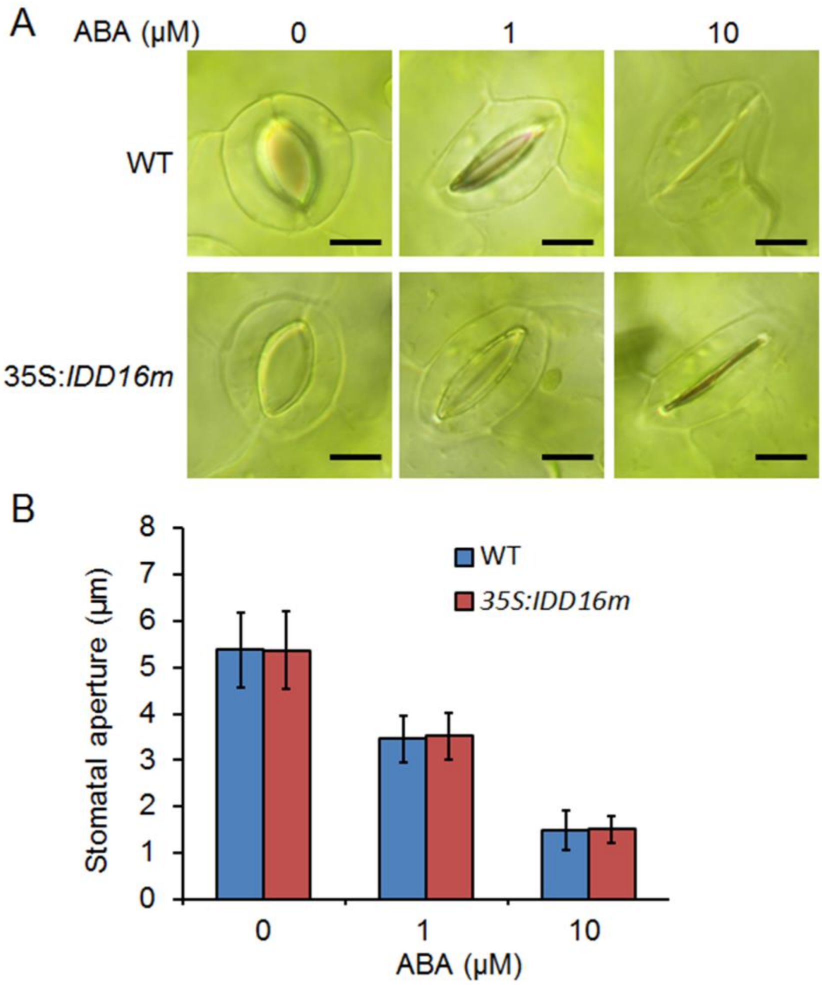

Supplemental Figure 9 35S:*IDD16m* plants exhibited similar stomatal aperture in response to ABA treatment compared with wild-type plants.

(A) Comparison of stomatal aperture in response to ABA treatment. (B) Stomatal aperture. Values are means ± SE from three biological replicates, and each replication with 30 stomata from leaves of three different plants.

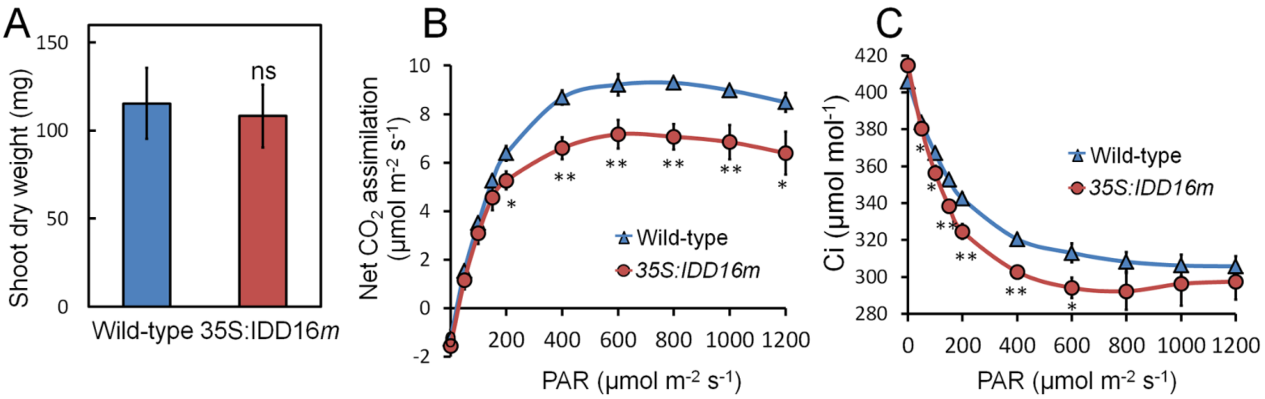

Supplemental Figure 10 Growth and carbon assimilation of 35S:*IDD16m* plants were not affected under normal growth condition.

(A) Shoot dry weight was measured at the completion of the WUE experiment. Values are means ± SE from four biological replicates. The data are from three replicates, with each replicate include more than 20 plantlets. **(B)** Net CO_2 assimilation and **(C)** internal CO_2 concentration(Ci) were determined on individual leaves of 5-week-old wild-type (Col-0) and 35S:*IDD16m* plants using a Li-Cor 6400 gas exchange system, values are the Mean ± SE (n = 4). Asterisks indicate statistical significance from the WT based on Student's t test; ** P < 0.01, * P < 0.05.

Table S1. Primers used in this study

	Primers	Sequence	Annotation
Primers for transgenetic plants			
	IDD16-F	CACCATGATACATTACGAACAAAACA	For the CDs of *IDD16*.
	IDD16-R	TCTCGCATTCTCCTTCAGT	
	*IDD16*i-F	GGGGACAAGTTTGTACAAAAAAGCAGGCTCACCATCCGGCAACCTCA	DNA fragments for *IDD16* silencing.
	*IDD16*i-R	GGGGACCACTTTGTACAAGAAAGCTGGGTATGGATAGTTGAAGTTCGAGGC	
	*IDD16*pro-F	AACTGCAGAAAGCAATAACCTAGATGAC	For the 2,621 bp promoter of *IDD16*.
	*IDD16*pro-R	GACTAGTTGATGCCAAGAAGAAGATCG	
Primers for qPCR			
AT3G13920	*eIF4a*-F	TGACCACACAGTCTCTGCAA	
	eIF4a-R	ACCAGGGAGACTTGTTGGAC	
AT1G25250	q*IDD16*-F	CAATGCTTCATCAGCTCCTTTC	
	q*IDD16*-R	CGTTCGCTCTCTCCTTTGTTA	
AT5G53210	q*SPCH*-F	ATCATAGGAGGAGTTGTGGAG	
	q*SPCH*-R	TAGAACAGGCGGTGAAGGAC	
AT3G06120	q*MUTE*-F	CGATCATCGGAGGAGTGATAGA	
	q*MUTE*-R	AAGGGAAAGATGGTCGGTTTAG	
AT3G24140	q*FAMA*-F	GAGCTCGAGCAACTCCTACAAT	
	q*FAMA*-R	GAAGTCGTTGTCGTTGTCATGT	
AT1G80080	q*TMM*-F	TCCTTCACCTAGAGGGCAATAA	
	q*TMM*-R	ACGGTACTGGTCCTGTCAGACT	
AT1G04110	q*SDD1*-F	AGCGGAATCACTGCTCTTATC	
	q*SDD1*-R	GCTGGTTTGTTACCATCCTTTATC	
AT2G20875	*qEPF1-F*	ATGCCGTCTTGTGATGGTTAG	
	qEPF1-R	TCAAGGGACAGGGTAGGACTT	
AT1G34245	*qEPF2-F*	TTTGGTCGTTAACTCCATTCG	
	qEPF2-R	ATCCGGTAAGCTTGATCCTGT	
At5g60880	q*BASL*-F	TCCTCTAGACGGAGATGAAGATGG	
	q*BASL*-R	TGGTGGGCTTAGGCTGAGTTTC	
At4g31805	q*POLAR-F*	TGAGTCACAAAGCAGAGAGTCACC	
	q*POLAR-R*	TCCATTTGCATTCGCAGGTTTGTC	
AT3G26744	q*ICE1*-F	TCTTTGCCTCCAACTTCATC	
	q*ICE1*-R	CACTGCTCTTCCTTCCCTTA	
AT1G12860	q*ICE2*-F	GTGTCCATCTTCCTCCTTGC	
	q*ICE2*-R	ACAGCTAATCACCGCTTGTT	
AT1G63700	q*YDA*-F	CACCATGAGATCACTGGACATT	
	q*YDA*-R	GCCATGTTTTAATCCTTTCTGC	
AT4G12970	q*STOMAGEN-F*	TGTAGTTCAAGCCTCAAGAC	
	q*STOMAGEN-R*	TTGATAGGGTCATTTCCTTC	

Table S1. Cont.

	Primers	Sequence	Annotation
AT2G26330	q*ERECTA*-F	GATAATGTCAAAGACGGGGAAC	
	q*ERECTA*-R	GGAAAACTTTCTTCACCACACC	
At5g62230	q*ERL1*-F	CGCATAACTTGCGGGAATTTG	
	q*ERL1*-R	AGTCCTTGTGCAGCTCCAACC	
AT5G07180	q*ERL2-F*	GCTGTGGATAACGAGGCCAAC	
	q*ERL2-R*	CATGGTGGGTCTCTCCAAAGG	
AT3G12280	q*RBR*-F	CAGATGGCTTGACCTACTTTGA	
	q*RBR*-R	CCTCTCATCAAGTTCGCCTTTA	
AT1G04710	q*PKT4*-F	ACGAGTTGCTTGCCTCTGTA	
	q*PKT4*-R	CACTGTCTGTTCACGGTTCTG	
AT3G48750	q*CDKA1*-F	GCCAAAAGCCCTTATTTCCT	
	q*CDKA1*-R	GTTACCCCACGCCATGTATC	
AT3G45640	q*MPK3*-F	TGACGTTTGACCCCAACAGA	
	q*MPK3*-R	CTGTTCCTCATCCAGAGGCTG	
AT1G51660	q*MKK4*-F	TCTTCCTCTCCCACCTACTTC	
	q*MKK4*-R	TGGAGCTGTTAGTGTTCGTTG	
AT1G68130	q*IDD14*-F	GGGACACCGGATCCGG	
	q*IDD14*-R	GTCTTGGTGCTCTATGAAACTTTCC	
AT2G01940	q*IDD15*-F	CCACAAACACATCCACTCAGA	
	q*IDD15*-R	TCTGGTCTCTTTGAAACCCTTG	
Primers for ChIP-qPCR			
	P1-F	ACTATGTCCGCTAAATCA	Used for detect the enrichment of regions 1, 2, 3, 4 of the *SPCH* gene.
	P1-R	ATGAAACATCAGCAATTAGA	
	P2-F	TAATTTGGGCTAGAAACAAAGC	
	P2-R	AGAAGATGGAGCCAAGAC	
	P3-F	ATGCAGGAGATAATACCG	
	P4-F	ATCATAGGAGGAGTTGTGGAG	
	P4-R	TAGAACAGGCGGTGAAGGAC	
At3g18780	ACTIN2-F	CGTTTCGCTTTCCTTAGTGTTAGCT	To detect the enrichment of *ACTIN2*, as internal control.
	ACTIN2-R	AGCGAACGGATCTAGAGACTCACCTTG	
AT3G30720	QQS-F	TCTGCAATTATGTAAACATA	Used for detect the enrichment of the fragment of *QQS* gene, as a positive control.
	QQS-R	ACAAATCAAAGGCCAATATC	
